# Longitudinal ultrasound imaging and network modeling in rats reveal sex-dependent suppression of liver regeneration after resection in alcoholic liver disease

**DOI:** 10.3389/fphys.2023.1102393

**Published:** 2023-03-09

**Authors:** Benjamin K. Barnhart, Toshiki Kan, Ankita Srivastava, Corinne E. Wessner, John Waters, Manju Ambelil, John R. Eisenbrey, Jan B. Hoek, Rajanikanth Vadigepalli

**Affiliations:** ^1^ Daniel Baugh Institute for Functional Genomics/Computational Biology, Department of Pathology and Genomic Medicine, Thomas Jefferson University, Philadelphia, Pennsylvania; ^2^ Department of Radiology, Thomas Jefferson University, Philadelphia, Pennsylvania

**Keywords:** ultrasound, liver regeneration, partial hepatectomy, alcohol, computational modeling, systems biology

## Abstract

Liver resection is an important surgical technique in the treatment of cancers and transplantation. We used ultrasound imaging to study the dynamics of liver regeneration following two-thirds partial hepatectomy (PHx) in male and female rats fed *via* Lieber-deCarli liquid diet protocol of ethanol or isocaloric control or chow for 5–7 weeks. Ethanol-fed male rats did not recover liver volume to the pre-surgery levels over the course of 2 weeks after surgery. By contrast, ethanol-fed female rats as well as controls of both sexes showed normal volume recovery. Contrary to expectations, transient increases in both portal and hepatic artery blood flow rates were seen in most animals, with ethanol-fed males showing higher peak portal flow than any other experimental group. A computational model of liver regeneration was used to evaluate the contribution of physiological stimuli and estimate the animal-specific parameter intervals. The results implicate lower metabolic load, over a wide range of cell death sensitivity, in matching the model simulations to experimental data of ethanol-fed male rats. However, in the ethanol-fed female rats and controls of both sexes, metabolic load was higher and in combination with cell death sensitivity matched the observed volume recovery dynamics. We conclude that adaptation to chronic ethanol intake has a sex-dependent impact on liver volume recovery following liver resection, likely mediated by differences in the physiological stimuli or cell death responses that govern the regeneration process. Immunohistochemical analysis of pre- and post-resection liver tissue validated the results of computational modeling by associating lack of sensitivity to cell death with lower rates of cell death in ethanol-fed male rats**.** Our results illustrate the potential for non-invasive ultrasound imaging to assess liver volume recovery towards supporting development of clinically relevant computational models of liver regeneration.

## Highlights

• Liver resection is necessary for living-donor transplants and potential treatment of liver cancer, but better tools are needed to assess and predict recovery.
• Longitudinal non-invasive imaging and computational modeling were used to assess the physiological mechanisms of liver regeneration in chronic ethanol-fed male and female rats, as well as pair-fed controls.
• Blood flow to the remnant liver increased for all animals in both the portal vein and hepatic artery, while chronic ethanol feeding led to decreased liver volume recovery exclusively in male rats.
• The dynamics of ethanol-mediated suppressed regeneration was best modeled by a lower metabolic load, without dependence on the cell death sensitivity.
• Immunohistochemical analysis of pre- and post-resection liver tissue validated the results of computational modeling by associating lack of sensitivity to cell death with lower rates of cell death in ethanol-fed male rats**.**

## Introduction

The healthy liver is capable of regenerating in response to injury or surgical resection. As the gold standard of treatment for liver tumors, liver resection requires visualization of the neoplasm and confidence that there will be enough tissue to regenerate a healthy organ (Yu, 2016). Liver resection is increasingly employed in living donor liver transplantation, which requires similar precise consideration of the living donor organ size and health (Miller et al., 2017). However, many liver diseases have been shown to impair normal liver regeneration, including chronic damage due to alcohol abuse (Taub, 1996). Liver regeneration after 70% partial hepatectomy is the most well-studied model of liver resection, with implications for many biological fields, including tissue repair, drug toxicity, cell pluripotency, and systems biology. Removal of left lateral and medial lobes causes waves of cellular proliferation and tissue growth in the remnant lobes to re-establish the pre-existing liver-to-body-weight ratio (Michalopoulos, 2017; Michalopoulos and Bhushan, 2021). After almost 90 years of experiments using this model, several questions persist about the biological processes governing this unique growth response, particularly those related to ethanol-induced suppression (Dippold et al., 2012, Hernández-Muñoz and Contreras-Zentella, 2019), and differences between male and female subjects (Tanaka et al., 1993; Morales-González et al., 1998). Adult rats adapted to Lieber-deCarli liquid ethanol diet that have been shown to have suppressed regeneration after 70% partial hepatectomy (Duguay et al., 1982; Diehl et al., 1990). Evidence also suggests that ethanol induces greater liver injury in females as compared to males (Meier et al., 2005), and this female-specific susceptibility has been seen in mice fed the National Institute of Alcohol Abuse and Alcoholism model of chronic-binge ethanol intake (Fulham and Mandrekar, 2016), and Wistar rats fed 4 weeks by intragastric enteral protocol (Kono et al., 2000). However, little is known about the interaction of chronic ethanol intake and liver resection in female rodents.

Magnetic resonance imaging (MRI) or computerized tomography (CT) can be used to image the liver (Noureddin et al., 2013; Jayakumar et al., 2019; Liu et al., 2019), but are not ideal for all patients due to radiation or high-density contrast agents, as well as high cost and lack of widespread availability (Clarke et al., 1989). In this regard, ultrasound for diagnostic imaging could be particularly advantageous in comparison to magnetic resonance imaging (MRI) or computerized tomography (CT) protocols due to lack of radiation or contrast agent, low cost, and portability (Mainenti et al., 2015). Ultrasound imaging is an alternative non-invasive approach to generate images and real-time visualization of tissues and internal organs based on high-frequency sound waves interacting with the living tissue. Ultrasound can measure organ stiffness as well as the rate of blood flow through elastography and doppler technologies, respectively (Hoskins, 2011; Choi et al., 2017; Wei and Song, 2020). Recently, ultrasound has been combined with photoacoustic imaging to detect tissue hypoxia in multiple internal organs (Gerling et al., 2014, Hysi et al., 2020, Karmacharya et al., 2020).

These non-invasive methods can quantify even minute longitudinal changes with respect to the entire liver during regeneration and may identify key characteristics of ethanol-induced impairment of regeneration. In addition to advances in ultrasound technology, liver resection is specifically poised to benefit from developments in computerized liver modeling. Efforts to develop a multi-scale computational model of liver regeneration seek to aid surgical risk-assessment and predict post-surgery liver function (Christ et al., 2017). Previous studies have collected organ-scale volume data to this end in wild-type mice (Hockings et al., 2002; de Graaf et al., 2011; Will et al., 2017; Zafarnia et al., 2019). A previous study also provided longitudinal liver volume data from CT of patients that have undergone liver resection to a varying degree, which was used to develop an empirical model of volume recovery dynamics (Yamamoto et al., 2016).

We have previously developed computational models of liver regeneration to describe multiple modes of the recovery process spanning from a normal response to suppression and total failure (Cook et al., 2015; Verma et al., 2018; Verma et al., 2019). These modeling studies demonstrate that detailed dynamics of liver regeneration can be replicated *in silico* by tuning a limited number of molecular and physiological parameters. It is also possible to fit this model to individual-specific longitudinal volume recovery data and gain an understanding of the biological mechanisms that would best describe variation between individual recovery rates. We intend to use this computational model to infer biological mechanisms underpinning the ethanol-induced impairment of liver regeneration after partial hepatectomy (PHx), with particular interest in the parameters that represent hepatocyte metabolism and cell death. These unmeasured physiological aspects represented as constant values in model simulations are highly impactful to regenerative outcomes.

A key feature of the computational models of liver regeneration is the accounting of the physiological stimulus that drives the response to PHx, represented in the model as a “metabolic load” on the liver. In addition to driving the initial signaling, this parameter is also considered as influencing cell injury and death in the early response post resection. Previous modeling studies demonstrated that the regeneration response was highly sensitive to the value of the metabolic load parameter, with low values leading to suppressed regeneration and very high values yielding liver failure post PHx (Cook et al., 2015; Verma et al., 2018; Verma et al., 2019). When the model was previously fit to regeneration data from various species, M correlated with average body mass of the organism (Cook et al., 2015). This parameter stands in for the biological need to maintain hepatocyte function and remains constant during simulation of regeneration. During resection, the metabolic load per hepatocyte is increased, inducing hepatocyte proliferation to compensate (Periwal et al., 2014). Thus, M functions as a set point for the organisms need for liver mass, and a steady driver to induce tissue growth after resection. Another parameter of interest due to model sensitivity is the cell death rate constant (Kcd). Kcd is a constant value which in part determines the likelihood that a hepatocyte will enter apoptosis in the computational model. In this way, Kcd represents the extent to which liver resection results in cell death. To examine the impact of chronic ethanol feeding on liver regeneration, in terms of these model parameters, we fit longitudinal liver volumes to the output liver mass of the computational model and performed immunohistochemistry assays to validate the results. Understanding impaired regeneration in terms of our biologically relevant model parameters maximizes the value of our non-invasive measures, giving insight into the underlying physiology of these animals.

We used a multi-modal ultrasound protocol to longitudinally observe liver resection in male and female rats that had adapted to chronic ethanol intake. Over a period of 2 weeks post-resection, we tracked liver volume, blood flow through both the right portal vein and main hepatic artery, stiffness, and tissue oxygen saturation. While testing the hypothesis that ethanol-induced suppression of regeneration would coincide with organ-scale physiological changes, we also sought to understand ethanol-impaired regeneration in the context of a mathematical model of liver regeneration. The rate of volume recovery in each animal was compared to simulated recovery using a well-established model (Cook et al., 2015), and immunohistochemistry was used to associate insights with changes in protein levels after PHx. By comparing observed data to simulated data with respect to the most sensitive model parameters, we provide a theoretical basis for future developments on risk assessment in clinical contexts.

## Materials and methods

### Animal model and dietary treatments

All animal studies presented here were conducted in accordance with the protocols approved by the Institutional Animal Care and Use Committee. The Lieber-DeCarli liquid ethanol diet is the standard treatment to assess chronic alcohol intake in rats (Lieber and DeCarli, 1982). Forty-three adult rats (23 male and 20 female) Sprague-Dawley rats (Charles River, Wilmington, MA) were fed the Lieber-DeCarli diet *ad libitum,* consuming 36% of calories from ethanol for 6–7 weeks (Bio-Serv, Frenchtown, NJ). As a control, a littermate was fed a liquid, zero-alcohol, calorie-balanced diet, with carbohydrates as the source of compensatory calories.

Animals arrived between 45–55 g as littermate pairs, and were allowed access to standard chow and water until reaching 120 g. At this time, animals on the liquid diet were introduced to the diet over the course of a week, first receiving the control diet for 2 days. The larger of the littermate pairs were then fed the ethanol liquid diet with 1/3 the total ethanol added for 2 days, followed by a 2/3 ethanol diet for an additional 2 days. The next day, the standard diets were given to each of the littermate pairs and continued for 6–7 weeks. An additional group was supplied solid rodent chow and water *ad libitum.* Animals were maintained on a 12:12-h light-dark cycle throughout the experiments.

### Liver resection

The left lateral and medial lobes (LLM) were removed during a 70% partial hepatectomy (PHx). This surgery is highly reproducible due to the simplicity of its techniques and high tolerability in rats (Higgins and Anderson, 1931; Crumm et al., 2008). Rats are sedated by 5% isofluorane in oxygen and maintained by 3% isofluorane. Surgery is performed on a heated pad to maintain body temperature. The left lateral and medial lobes are ligated with a silk suture. Polypropelene sutures are used to close the incision, as veterinary staples interfere with the ultrasound imaging. All animals in this study were maintained under isofluorane sedation between surgery and ultrasound imaging at the 20-min time point. The remaining right and caudate lobes were allowed to regenerate. Importantly, animals on the liquid diets described above were allowed access to diet up to the point of isofluorane induction, and throughout the recovery period.

### Ultrasound imaging

Ultrasound imaging was performed prior to PHx, immediately following PHx, and each subsequent day during recovery. Multiple imaging techniques were employed during a continuous isoflurane sedation. Each animal was imaged for approximately 20 min under anesthesia (induced by 5% isofluorane and maintained by 3% isofluorane). Average time of sedation was not significantly different between any experimental groups. After completing all the imaging acquisition (described further below), the rats were placed under a heat lamp until 10 min after waking from the anesthesia.

### Liver volume quantification

Liver volume was measured with the VEVO 2100 (VisualSonics, Toronto, Canada) high-frequency ultrasound system with a motorized-step linear-array transducer transmitting at a center frequency of 21 MHz. During each scan, the transducer was moved 30 mm along the longitudinal axis, with a step size of 0.07 mm and imaging depth of 30 mm. Because the width of the right hepatic lobe exceeded that of the transducer, two side-by-side scans were combined using the main portal vein and intra vena cava as anatomic landmarks.

### Portal and hepatic artery flow rate measurement

The main portal vein and hepatic artery were visualized in a longitudinal plane using the VEVO 2100 System (VisualSonics, Toronto, Canada) with a 21 MHz linear transducer at an imaging depth of 30 mm. Color Doppler was optimized for each animal by adjusting the velocity and gain. After color Doppler images were acquired, the pulse-wave Doppler was acquired to collect velocity measurements (mm/s). For each animal, each vessel diameter was measured at the same location. The velocity measure was multiplied by the cross-sectional area of vessel calculated as 𝛑 x (diameter/2)^2^ to calculate volumetric flow rate, which was then divided by liver volume for normalization (ml/min/cm^3^). In some cases, difficulty in visualizing the smaller hepatic artery and time restrictions due to length of sedation led to missing data for flow rates through the hepatic artery.

### Elastography

Liver stiffness was measured using shear wave elastography on an Aplio i800 (Canon Medical Systems, Tustin, CA, United States) ultrasound system with a curved-array transducer transmitting at 15 MHz. Like B-mode imaging, an imaging depth of 30 mm was used. All measurements were taken from the right lobe, keeping a consistent angle and pressure on the transducer. Three circular regions of interest (2-inch diameter) were used from a single image to calculate an average velocity.

### Photoacoustic imaging

Photoacoustic (Vevo 2100 LAZR) imaging was performed to quantify oxygen saturation in the right anterior hepatic lobe of all rats. Three 30-s videos were captured for each animal at each time point. One video with the least interference (best signal) was selected and a region of interest was drawn at the anterior surface of the right hepatic lobe, with an area of approximately 3 mm by 10 mm. The data was exported from the VEVO 2100 to VEVOlab software, and the average oxygen saturation was recorded.

### Statistical analysis

Statistical significance was calculated based on an independent measures ANOVA, where sex, diet and time were considered as grouping variables. Error estimates were based on a factorial linear model:

Measured variable∼sex+diet+time+sex:diet+sex:time+diet:time+sex:diet:time (1)

All tests were performed in the R programming language version 4.0.3 (R Core Team, 2020) using the *lm* function in the *stats* package version 4.0.3 (R Core Team, 2020) and the two functions *anova_test* and *emmeans_test* from the *rstatix* package version 0.7.0 (Kassambara, 2021). The *emmeans_test* function adjusts the raw *p*-values using a Bonferroni post-hoc test to determine significance. All the values given in the results are represented as mean ± standard deviation.

### Overview of computational modeling

The computational model used in the present study was originally developed in Cook et al. (2015). Briefly, the model is comprised of ordinary differential equations that estimate the relative proportion of hepatocytes in a regenerating liver. The initial parameters (constants values) assume the remnant fraction (30%, for a 70% partial hepatectomy) of hepatocytes are quiescent (Q) before transitions to the primed (P) and replicating (R) states occur. In the model simulations, the total liver volume is computed as (Q + G*(P + R))+epsilon) representing the sum of hepatocytes in different cell states.

The hepatocyte state transitions and proliferation are stimulated through multiple pathways accounted in the model as a physiological stimulus representing the overall metabolic load (M). The model also integrates cell growth and cell death equations to tune the overall rate of mass recovery. A key component of the cell death equations is the constant rate of cell death (Kcd), which represents the likelihood a cell enters apoptosis during the replication cycles induced by resection. The full set of equations for the model are provided in Supplementary File S1 and the parameter values are included in Supplementary Table S1.

### Matching experimental data to computational model parameters

The original model of Cook et al. (2015) was implemented in MATLAB software (Natick, MA). In the present study, the model code was translated into the R programming language (R Core Team, 2020). The model was simulated for sequential values of a combination of the parameters M and Kcd. For M, 100 sequential values were chosen in a linear range of 5–30, while 100 values of Kcd were selected from a logarithmic sequence of range 5 × 10^−4^ to 0.5, for a total of 10,000 simulations to be compared against experimental data.

For each animal, measured liver volume was subtracted from simulated liver volume as a residual at each time point of experimental data. A log-likelihood measure was calculated from these residuals in an unbiased manner as follows:

$$\mathrm{Log}\;Likelihood=\overset{n}{\underset{1}{\sum }}\log\left(p\left(x\right)\right)$$ (2)

where *x* is the residual at each of the *n* time points, *p* represents a Gaussian probability distribution:

$${p\left(x\right)=e}^{\left.-(x-\mu {)}^{2}/(2\sigma^{2}\right)}/\sigma \sqrt{2\pi }$$ (3)

where σ is the standard deviation of the residuals *x*_*1*_, x_*2*_,…x_*n*_, and the average of the residuals, μ, is set to 0. The final equation below was used to weight the last time point, so that log-likelihood was affected to a greater extent by the volume recovery data at the end of the experimental time series.

$$\mathrm{Log}\;Likelihood=\log(p(5x_{n})+\overset{n-1}{\underset{1}{\sum }}\log\left(p\left(x\right)\right)$$ (4)

The above weighted log-likelihood was calculated against all 10,000 simulations for each animal with at least five liver volume measurements. These likelihoods within the designated parameter space were used for comparing the fit to simulated data across the four experimental groups (male and female X ethanol and pair-fed control). For the correlation analysis of model parameters and pre-surgery physiological measures, we used the optimal parameters obtained from maximizing unweighted log likelihood.

### Model reproducibility and credible practice

All simulation results and figures presented in the current work were repeated independently by a departmental colleague, not involved in the original modeling and simulation study, by using the code available on github for the present study. The independently repeated simulations results were in agreement with the results presented here. Parameter values in the code were cross-checked for consistency against the values provided in Supplementary Table S1. For the purpose of assessing reproducibility, all model files were uploaded to GitHub (https://github.com/Daniel-Baugh-Institute/LiverVolumeSimulations) and are also available as a supplement to the present manuscript. A self-assessment of the study for conformance to the Ten Simple Rules for Credible Practice in Modeling and Simulation in Healthcare was performed and the results are included in the supplement (Supplementary File S2).

### Immunohistochemistry staining

The liver tissue samples (∼2 mm diameter) were collected from excised liver tissue and locked in Tissue Embedding Cassettes and fixed in 4% Paraformaldehyde (PFA) (Electron Microscopy Sciences, Hatfield, PA, United States) for 24 h on a shaker. The cassettes were then washed three times in distilled water and placed in 70% ethanol (Decon Labs, Glennie Circle King of Prussia, PA, United States) at 4°C until paraffin embedding. The paraffin embedding and sectioning at 5 µm thickness was performed by the Translational Research/Pathology core facilities at Thomas Jefferson University. For immunohistochemistry, paraffin-embedded liver tissue sections were deparaffinized in Xylene (Sigma Aldrich, Burlington, MA, United States) and rehydrated in decreasing ethanol concentrations followed by distilled water. The heat-induced antigen retrieval was performed using 10 mM citrate Sodium Citrate buffer (pH = 6) (ab64214; Abcam, Cambridge, MA, United States) by heating the slides in a buffer solution of sodium citrate with 0.05% Tween 20 (BIO-RAD, Hercules, CA, United States) in the microwave for 15 min. The endogenous peroxidases were blocked using hydrogen peroxide (Abcam) for 15 min, followed by three washes of 1x PBST (1x PBS with 0.1% Tween-20) at 5 min intervals each. The sections were permeabilized with 0.2% Triton-x (LabChem, Zelienople, PA, United States for 20 min followed by two washes of 1xPBST at 5 min intervals each. The sections were blocked using 10% normal goat serum (ab7481; Abcam) for 1 h at room temperature. Blocking was followed by overnight incubation with the primary antibody at 4°C followed by three washes of 1x PBST at 5 min intervals each. Primary antibodies used in the staining included anti-Ki67 antibody (ab279653; Abcam) used at (1:1000), M30 CytoDEATH antibody (12140322001; Roche, San Francisco, CA, United States) (1:25), Hepatic Sinusoidal Endothelial Cells Antibody (NB110-68095; Novus Biologicals, Centennial, CO, United States) (1:200) or Aspartate Aminotransferase Antibody (NBP2-57518; Novus Biologicals) (1:1000) dilution. Secondary antibody incubation and signal detection were performed using the DAB chromogen method (ABC) Detection IHC kit (ab62464; Abcam). Biotinylated Goat Anti-Polyvalent was applied on the tissue sections for 10 min at room temperature, followed by three washes of 1x PBST at 5 min intervals each. Next, Streptavidin Peroxidase was applied to the sections for 10 min at room temperature, followed by three washes of 1x PBST at 5 min intervals each. DAB reagent was prepared by combing 30 µL of DAB Chromogen to 1.5 mL of DAB substrate. DAB reagent was applied to the sections for 3–10 min. The reaction was stopped by cold tap water depending on the signal intensity. The sections were counterstained with Hematoxylin (ab245880; Abcam) for 5 min at room temperature; the slides were washed in cold tap running water until the water turned clear. The sections were then incubated in Bluing Reagent for 45 s. The slides were washed in distilled water. Dehydration was performed using increasing ethanol concentrations followed by xylene before mounting using Limonene Mounting Medium (ab104141; Abcam).

### Imaging and quantification of IHC slides

All IHC-stained slides were imaged for quantification at ×100 magnification (×10 objective and ×10 base magnification) with a resolution of ×1920 1440 (pixel size: 0.454 µm × 0.454 µm). For each slide, 10 non-overlapping images were collected to capture the majority of tissue in brightfield. Cell Profiler v. 2.4.1 (Stirling et al., 2021a) was used to process all images. Briefly, the brightfield images are split into channels specific to the DAB chromogen stain and hematoxylin. A Gaussian filter is used to smooth the hematoxylin channel before an intensity threshold is applied to remove background noise. Nuclei are segmented using the “IdentifyPrimaryObjects” module in Cell Profiler.

For the Ki67-stained images, size and shape filters are applied to the segmented nuclei to remove any noise, and the “ShrinktoObjectCenters” function is used to store all nuclei as their coordinates on each image. Separate channels of each image are saved. A separate Cell profiler pipeline is then used to create the SQL database used to run the machine learning app, Cell Profiler Analyst (Stirling et al., 2021b). The classifier tool was used to sort all nuclei into one of four categories: “DAB-Positive Hepatocytes,” “DAB-Negative Hepatocytes,” “DAB-Positive Non-Parenchymal Cells,” and “DAB-Negative Non-Parenchymal Cells.” About 3,000 nuclei were sorted before training and scoring all images. A Convoluted Neural Network of 12 by 12 neurons per layer was used.

For all other images, the segmented nuclei were used to infer cell cytosolic area before quantifying average DAB signal for all cells. The bright field image was converted to gray scale, smoothed and thresholded as above to remove noise. The segmented nuclei and total-tissue object were used as input for the “IdentifySecondaryObjects” module, which approximates a cell area around each nuclei using a gradient-based watershed. DAB signal was only considered in areas of the tissue that did not overlap with the segmented nuclei.

We show data for Ki67 and M30 for three animal pairs from each of the four experimental groups (Total N = 12: Male Carbohydrate n = 3, Male ethanol n = 3, Female Carbohydrate n = 3, and Female ethanol n = 3). For Got1 and Se-1, only 1 animal pair was used (Total N = 4: Male Carbohydrate n = 1, Male ethanol n = 1, Female Carbohydrate n = 1, and Female ethanol n = 1). Cell profiler pipelines used for this analysis are included as supplementary files.

### Fibrosis scoring of whole slide images of the liver tissue sections

For Picrosirius Red staining, the paraffin-embedded liver tissue sections were first deparaffinized and rehydrated. The slides with the liver tissue sections were incubated in Bouin′s solution (HT10132; Sigma Aldrich) overnight at 55°C and then cooled to room temperature. The slides were incubated in 0.1% of Picrosirius Red stain (365548; Sigma Aldrich) for 30 min at room temperature. The slides were washed twice in deionized water. Dehydration was performed using increasing ethanol concentrations followed by xylene and then coverslipped. Picrosirius Red stained liver tissue sections were scanned on an Aperio ImageScope digital slide scanner (Leica Biosystems, Wetzlar, Germany) to generate whole slide images (WSIs) at ×20 magnification. Using the Visiopharm image analysis software (Visiopharm Corporation, Westminster, CO), an Application Protocol Package (APP) was developed to accurately detect tissue within each WSI for further analysis. A separate, pre-trained APP conducted segmentation of the portal triad and central veins within the tissue using the convolutional neural network Deeplabv3+ (Beatson et al., 2020). Fibrosis was detected within and between segmented portal triads and sinusoidal spaces using the linear Bayesian method. After segmentation was completed, the Visiopharm APP generated scored each sample using the Brunt fibrosis staging system (Brunt et al., 1999). A score of 0 indicates no significant presence of fibrosis. The Picrosirius Red stained liver tissue sections were independently evaluated and scored for fibrosis by a gastrointestinal pathologist.

## Results

Liver volumetric recovery is impaired by chronic ethanol feeding in rats, with sex-dependent differences.

**FIGURE 1:**
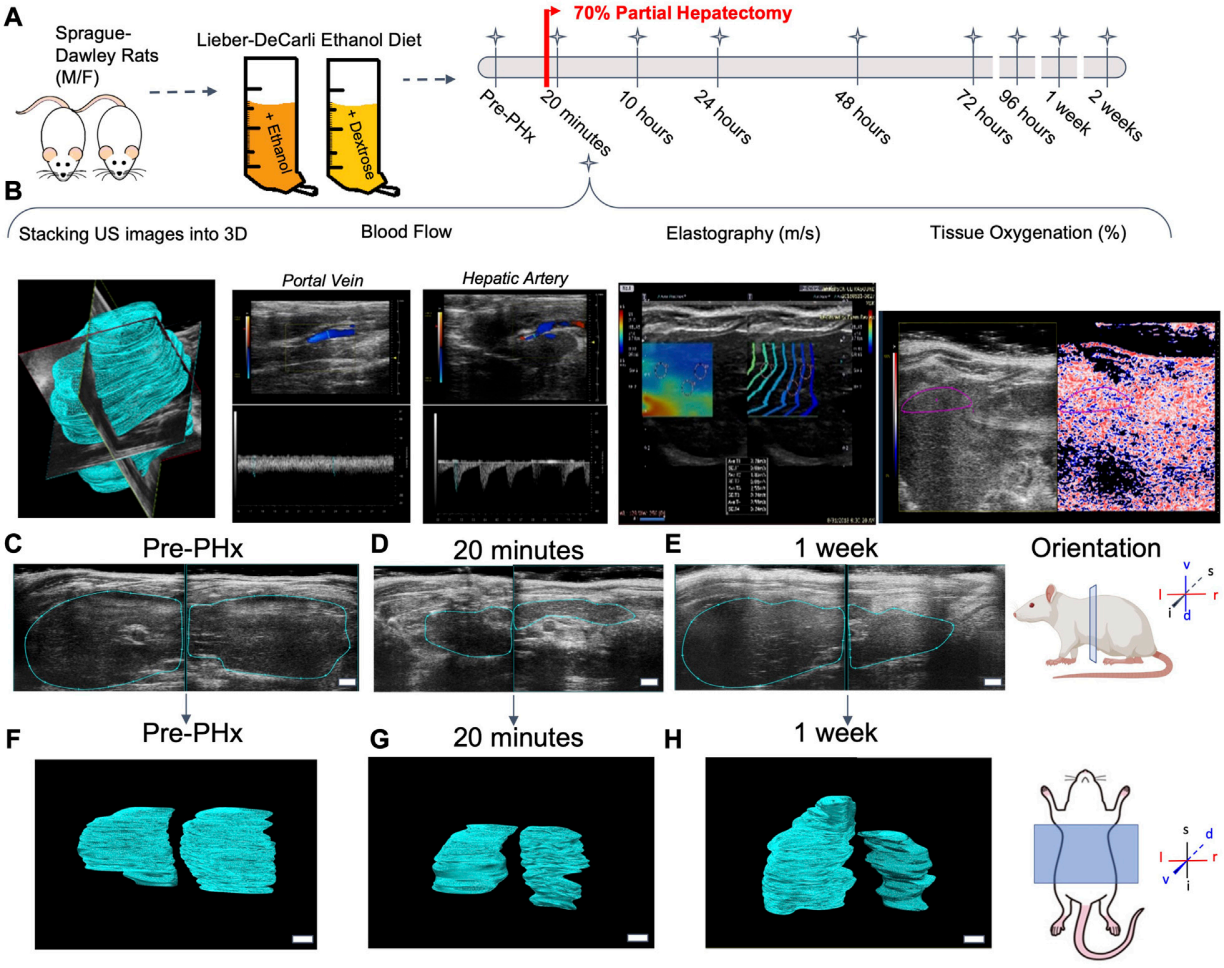
Longitudinal study of liver regeneration by quantitative non-invasive ultrasound imaging. **(A)** Schematic of the overall workflow. Briefly, adult male and female rats were fed Lieber-Decarli liquid ethanol diet or pair-fed calorie-matched liquid control diet. An additional group of male and female rats were fed solid rodent chow diet *ad libitum*. A 70% partial hepatectomy was used to stimulate liver regeneration. **(B)** The day prior to surgery, and at each successive time point indicated, the liver volume was measured by a motorized step-transducer. Volumetric blood flow rates, liver stiffness, and tissue oxygenation were also measured non-invasively at each time point. **(C–E)** Representative 2D transverse cross-sections of the liver from a female rat fed solid chow diet. Scale bar indicates 3 mm. **(F–H)** Complete 3D rendered liver volumes from the corresponding image stacks, viewed in frontal plane. Scale bar: 2 mm.

Liver regrowth after partial hepatectomy (PHx) has been shown to be impaired by chronic ethanol intake in the rat (Diehl et al., 1990). We used the same experimental model of chronic ethanol feeding, which uses an *ad libitum* liquid diet with 36% of calories from ethanol. Data collection occurred 24 h prior to surgery, and directly after PHx, animals remained under sedation for the initial post-PHx measurements. Longitudinal data collection occurred for all animals in a variable combination of the following time points post-PHx: 10, 24, 48, 72, and 96 h, 1 week, and 2 weeks (Figures 1A, B). At least five data collections were done for each animal, with few exceptions. See Supplementary File S3 for complete details on each animal. Liver volume was measured using a motorized-step transducer in B-mode ultrasound imaging. The liver was segmented manually by processing individual frames of transverse image stacks and rendered into volumes for quantification (Figures 1C–H).

**FIGURE 2:**
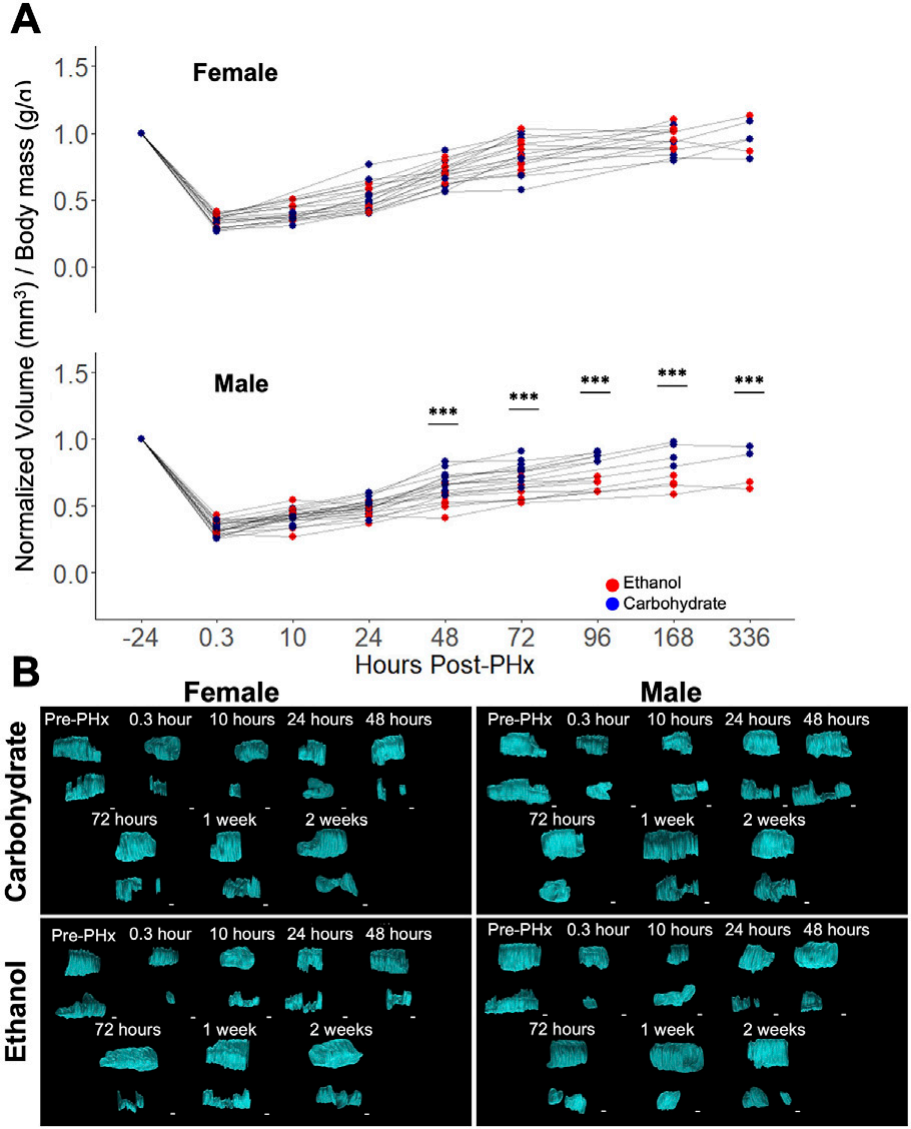
Longitudinal study of liver volume measured by ultrasound 3D reconstruction using a motorized-step transducer in B-Mode. **(A)** Liver-volume-to-body-mass ratio, normalized to pre-surgery values (−24 h post-PHx). Thin grey lines connect data points corresponding to an individual animal. *** indicates *p* < 0.001. **(B)** Representative 3D reconstructions of the liver at various time points in four animals. Each time point has two volumes due to the limited size of area visible by a single transducer (see Figure 1B). Liver volumes viewed along the transverse plane, where the animal head is to the right, tail to the left. Top volume moved from behind lower volume. See Supplementary Videos S1–S4 for the corresponding images with rotation. Scale bars: 2 mm.

Baseline values of liver volume were compared to longitudinal measurements after 70% PHx. A significant impairment in volume recovery was observed in ethanol-fed males (Figure 2A). However, no difference was seen between the female diet groups and male controls (Figure 2A). Significant differences in male rat liver volumes were seen beginning at 48 h post-PHx and continued for up to 1-week post-PHx (*p* < 0.001). All female rats and male control rats recovered to nearly 100% of their original liver volume by 1 week (Female Control: 91.54% ± 10.39%, Female ethanol: 97.13% ± 8.37%, Male Control: 90.04% ± 8.68%, mean ± s.d.; Figure 2A). By contrast, the liver in male ethanol-fed rats had recovered to only 65.93% ± 5.91% of the pre-surgery liver volume. Examination of the 3D reconstructed volumes suggests that the ethanol-mediated reduction in recovery was not uniform across the measured extent of the liver, likely attributable to the differences between right lobe and left caudate lobe (Figure 2B; Supplementary Videos S1–S4). However, the limited width of the ultrasound transducer necessitates two separate iterations of data acquisition per animal for covering the full extent of the liver, which makes it difficult to definitively conclude about the lobe-specific variation of recovery at this level of analysis.

**FIGURE 3:**
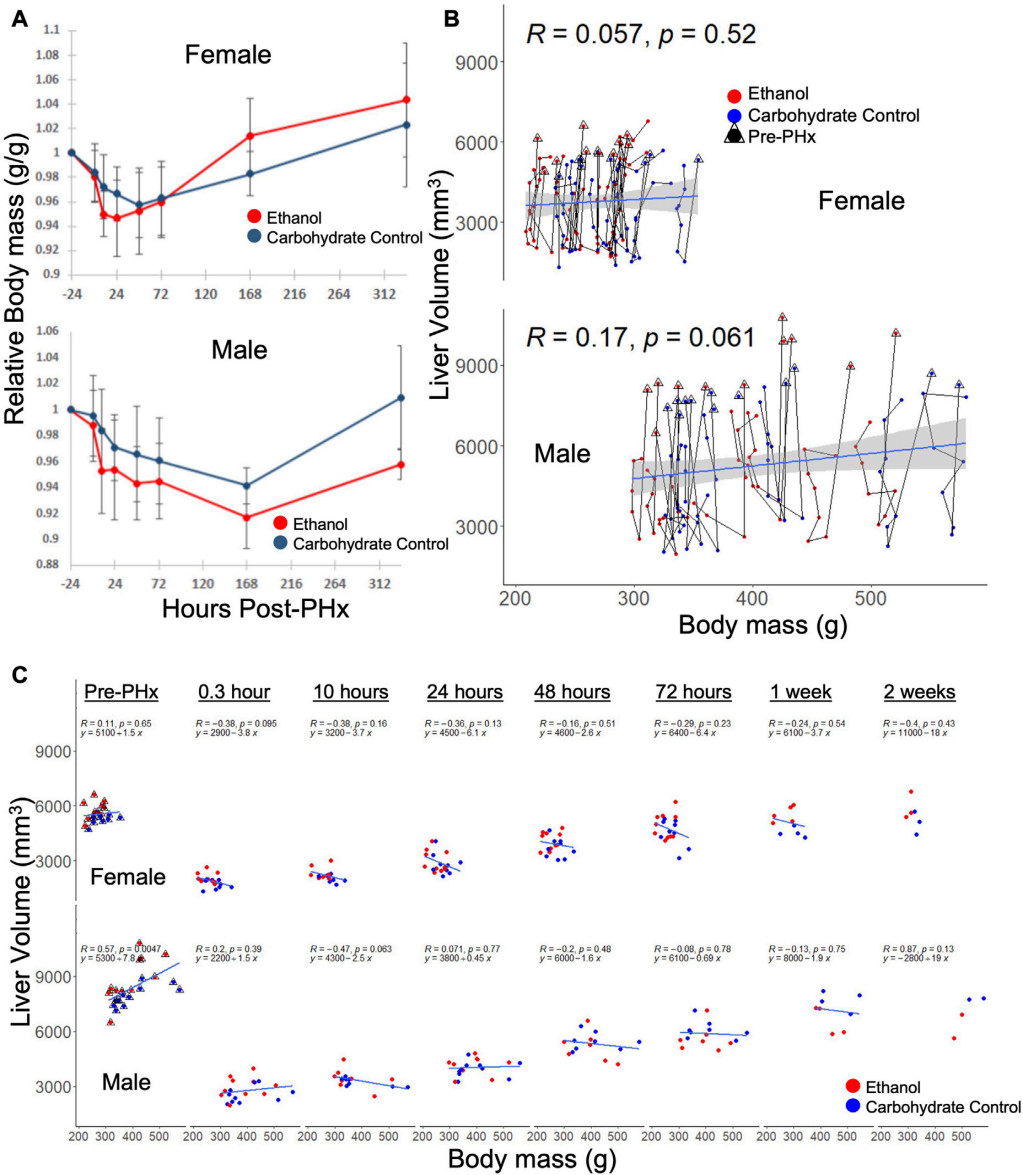
Relationship between body mass and liver volume during recovery from 70% PHx. **(A)** Average change in animal mass during recovery, red indicates ethanol-fed animals, while blue indicates control animals. PHx indicated by dashed red line. Error bars represent standard error of the mean. **(B)** Individual animal traces showing changes in body mass relationship to liver volume. Data within triangles indicate pre-surgery (24 h prior to PHx) values and connect chronologically to data from the same animal. Blue lines show linear model (y ∼ x) calculated from the female and male data, respectively, where correlation (R) and significance (p) are shown. The grey area indicates standard error of the mean. **(C)** Data from **B** separated by time post-PHx. Blue lines are time-point-specific linear models as in **(B)**.

Although body mass was used to normalize the volume recovery data, there was some concern that sex-based differences in volume recovery may be due to differences in average body mass. To confirm the significant ethanol-induced difference in volume recovery between male and female rats, we examined the correlation between body mass and liver volume in each data set. In both male and female rats, there were no significant differences in relative body mass between diets at all time points, with the exception of male rats at 10 h post-PHx (p_adj_ = 0.017, all other p_adj_ > 0.09; Figure 3A). When considering male or female across all time points, liver volume did not correlate well with the body mass (Figure 3B). However, female rats with less body mass tend to recover liver volume more quickly (not significant, *p* = 0.095–0.65; Figure 3C). Taken together, these results support the conclusion that sex-based differences in liver volume recovery were not affected by differences in body mass.

### Impaired liver regeneration is associated with increased blood flow through the portal vein

**FIGURE 4:**
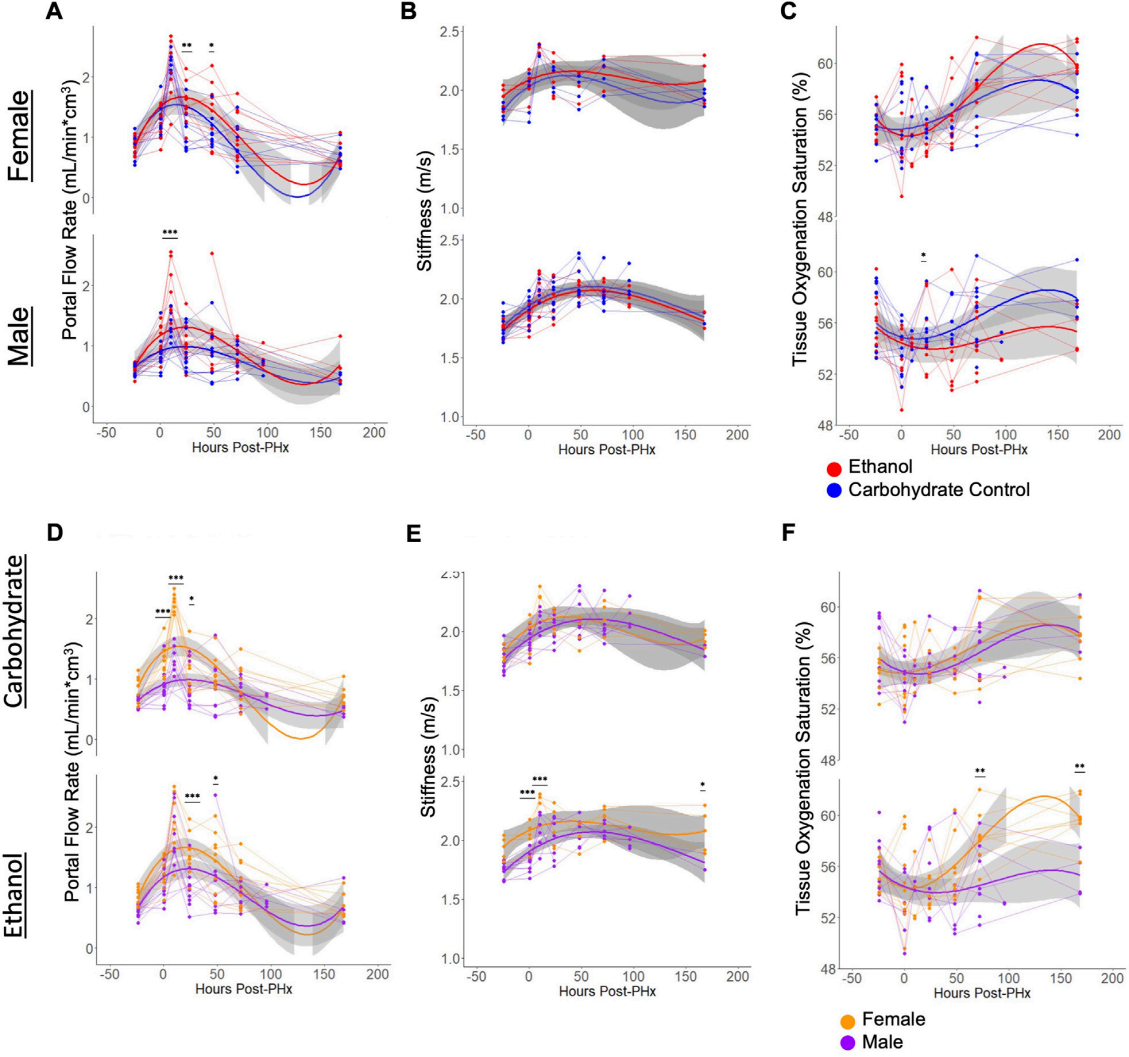
Physiological ultrasound measurements, with comparisons between male/female and ethanol-fed/control-fed animals. For all plots, light grey lines connect data from a common animal. Curved lines indicate polynomial models (y ∼ x + x^2^+ x^3^) of the data indicated by line color. Grey area indicates standard error of the mean. **(A, D)** Volumetric flow rate of blood through the main portal vein. The same data is shown in **(A, D)**, with different grouping. **(B, E)** Elastography measurement of liver stiffness. The same data is shown in **(B, E)**, with different grouping. **(C, F)** The proportion of hemoglobin bound to oxygen in blood of the liver tissue. The same data is shown in **C** and **F**, with different grouping. For all panels, *** indicates *p* < 0.001, ** indicates *p* < 0.01, and * indicates *p* < 0.05.

Flow through the portal vein and hepatic artery were measured by color doppler and multiplied by vessel diameters (measured simultaneously) to give volumetric flow rates. When normalized to the liver volume, increase in flow rates were observed for all animals immediately following PHx, and at 10 h post-PHx (Figures 4A, D). There was no significant difference between any of the experimental groups in baseline flow rates, and all animals showed an increase in portal flow in the first 10 h post-PHx. Male ethanol-fed rats had significantly elevated portal flow at 10 h post-PHx (ethanol Males: 1.92 ± 0.44, Control Males: 1.31 ± 0.22, mean ± s.d, p_adj_ = 1.1 × 10^−4^), but ethanol-fed females were similar to their control-fed counterparts at the same point in recovery (ethanol Females: 2.09 ± 0.48, Control Females 2.27 ± 0.16; mean ± s.d, *p* = 0.266). In order to assess the relationship between portal flow and volume recovery, the portal flow at 10 and 24 h was plotted against volume recovery at 1 week, but only poor correlations were found (R = 0.17, −0.27 and *p* = 0.41, 0.2, for 10 and 24 h, respectively; Supplementary Figures S1A, B). A weak correlation was also found between the portal flow rate and liver volume when considering all time points (R = 0.25, *p* = 4.4 × 10^−5^; Supplementary Figure S1C). Interestingly, flow through the hepatic artery had average increases up to 10 h post-PHx for all animals (Supplementary Figures S1E, F). Though varied in duration, PHx-induced increases in blood flow through both PV and HA had universally returned to pre-PHx levels after 1 week of recovery (Figures 4A, D, Supplementary Figures S1E, F).

### PHx induces an increase in liver stiffness that lasts a week post resection

Similar to portal vein blood flow, liver stiffness as measured by shear wave elastography showed increases for all rats at 10 h after PHx. The stiffness remained elevated until 1 week post-PHx (Figures 4B, E) and normalized to pre-surgery levels at that time. While there were no significant differences in stiffness between diet groups at any time point, ethanol-fed animals showed sex-specific differences prior to surgery (ethanol Males: 1.75 ± 0.06, ethanol Females: 1.93 ± 0.09, mean ± s.d, p_adj_ = 0.0066). and up to 10 h after PHx (ethanol Males: 2.07 ± 0.15, ethanol Females: 2.35 ± 0.04, mean ± s.d, p_adj_ = 0.0013). At 1-week post-PHx, ethanol-fed males returned to a stiffness below that of ethanol-fed females (ethanol Males: 1.82 ± 0.10, ethanol Females: 2.08 ± 0.18, mean ± s.d, *p* < 0.05). Also, there was a moderate correlation between portal vein blood flow rates and the stiffness measurements (Supplementary Figure S1D). We evaluated all the conditions for the presence of any fibrotic tissue. We performed Picrosirius red staining and independently assessed fibrosis by automated image analysis as well as by a pathologist. Our results indicate lack of fibrosis in all the conditions, suggesting that the stiffness differences we observed are not likely due to underlying changes in collagen deposition in the livers of the ethanol-fed animals (Supplementary Figure S2; Supplementary Table S2).

### Oxygenation of tissues shows PHx-dependent changes

There was high within-group variability in oxygenation compared to the other parameters measured using the non-invasive ultrasound approach. Male rats appeared to follow a pattern of decreased tissue oxygenation directly after PHx that resolved by 24 or 48 h of recovery. Most female rats also followed this pattern, though some showed increased oxygenation directly after surgery. The only significant difference between diet groups was seen in males at 48 h after PHx (ethanol: 53.53 ± 3.26, Carbohydrate: 55.91 ± 1.57, mean ± s.d, p_adj_ = 0.039). Among ethanol-fed animals, female rats had significantly elevated tissue oxygenation by 3 days post-PHx (ethanol Males: 55.08 ± 2.65, ethanol Females: 58.34 ± 2.06, mean ± s.d, p_adj_ = 0.0034), which sustained to 1 week post-PHx (ethanol Males: 55.42 ± 1.78, ethanol Females: 59.74 ± 1.70, mean ± s.d, p_adj_ = 0.0021; Figures 4C, F).

### Computational modeling predicted differential physiological stimuli controlling liver regeneration in ethanol-fed rats

**FIGURE 5:**
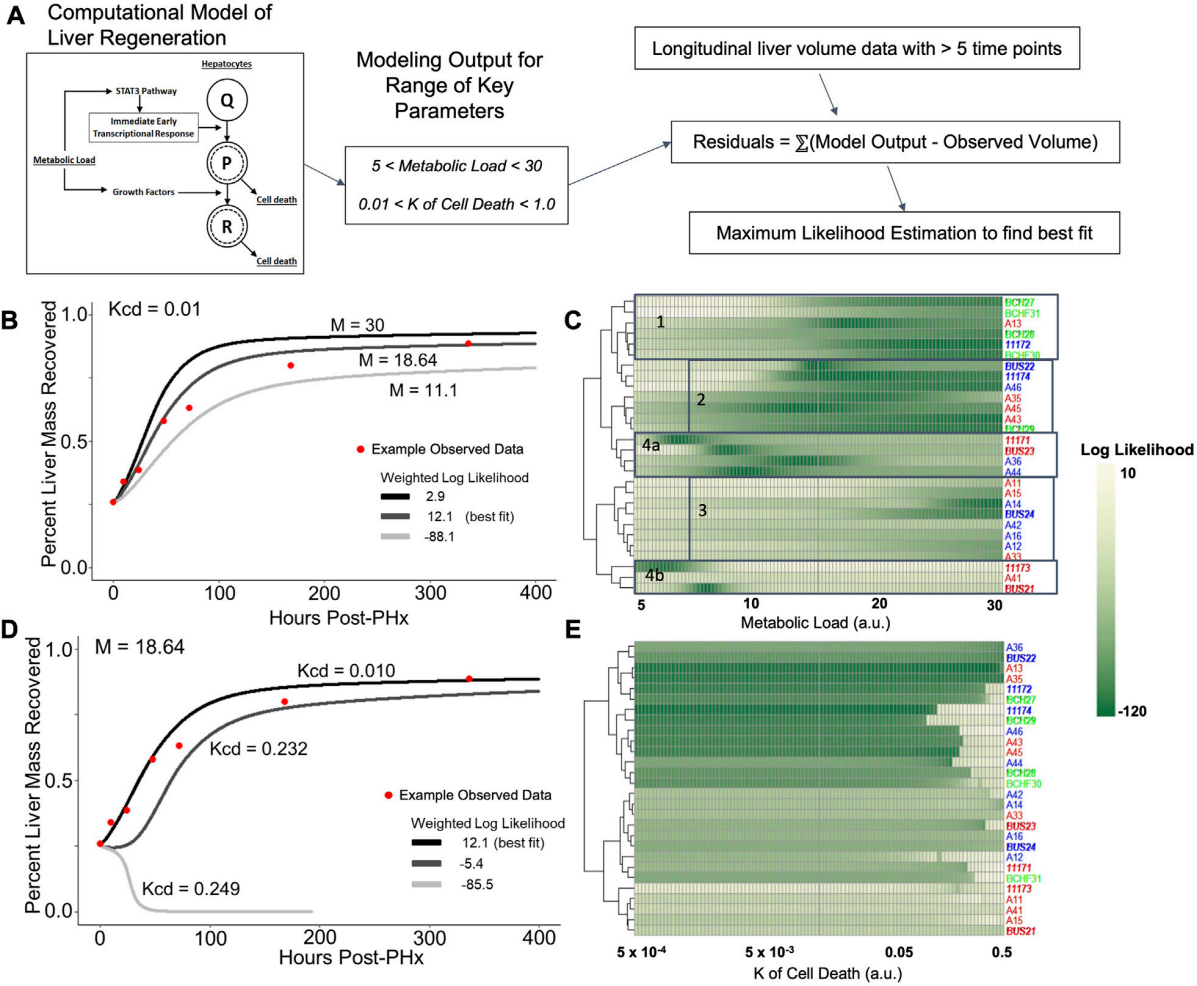
Log likelihood of residuals calculated from the difference between longitudinal liver volumes measured *via* ultrasound and simulated liver mass using a computational model of regeneration. **(A)** Schematic of the analytical pipeline. Simulated liver volume for a range of two parameters is compared to the observed data shown in Figure 2 by maximum likelihood estimation. **(B)** Fit of simulated-to-observed data as the metabolic load parameter (M) changes from 5 to 30 (arbitrary units) for each animal measured at least 1 week after PHx (minimum ultrasound measurements). Animal identifiers on the *y*-axis are color-coded to indicate diet: blue highlight indicates control-fed, red indicates ethanol-fed, and green indicates solid-chow-fed animals. Female animals are labeled in plain font, while males are italicized. **(C)** Log likelihood data for varied M for all animals. **(D)** Similar to B, with the K of cell death (Kcd) varied over a range of 5e-4 to 0.5 (arbitrary units). **(E)** Log likelihood data for varied Kcd for all animals.

The liver volume recovery time series data was compared to the predictions from a model of liver regeneration previously developed by our group (Cook et al., 2015). This model-fitting analysis reveals possible biological implications of the volumetric recovery data, by accounting for differences in experimental data by tuning biologically relevant parameters (Figure 5A). Previous work with the model highlighted several parameters that demonstrate high sensitivity in the model, where small changes to the constant values have large impact on simulated rate of recovery (Cook et al., 2015; Verma et al., 2018; Verma et al., 2019). The first parameter of interest was metabolic demand (M), a physiological stimulus that controls the rate of regeneration in the model. The value of M influences both the initial signaling stimuli as well as cell death, thus shaping the balance of regenerative and potential failure processes immediately post PHx.

The value of M was varied sequentially across 100 simulations, while all other parameter values were fixed to previously optimized values for rat liver volume recovery data (Figure 5B; Supplementary Table S1; Verma et al., 2019). For each animal with at least five volume measurements, log-likelihood was used to compare the observed data to all 100 simulations. Log-likelihood was calculated using the residual difference between the volume recovery estimate of the model and the observed volume recovery of each animal, to estimate a generalized measure of how well the given model-predicted liver volume dynamics matched the observed data. While most of the data was best fit by M values between 15–25, data from the ethanol-fed males were best fit by M values of ∼9 (Figure 5C). Within the control males and all females, there appeared to be three clusters of metabolic demand levels that gave the best fit (indicated by boxes in Figure 5C). Interestingly, strong correlations were found between the optimal metabolic demand for each animal and pre-PHx ultrasound observations, including liver volume (R = −0.65, *p* = 0.00021), portal vein and hepatic artery flow rates (R = 0.5, 0.48, and *p* = 0.0079, 0.02, respectively) as well as body mass (R = −0.6, *p* = 0.00072; Supplementary Figure S3).

In parallel, we simulated the model by introducing variations in another model parameter with a high sensitivity, the rate of cell death (Kcd; Figure 5D). We previously showed that the cell death parameters robustly predicted long-term results of liver recovery (Verma et al., 2019). Our results show that the animals with higher predicted metabolic demand levels (M values) also tend to yield a good fit for a wide range of Kcd values, while animals with lower predicted M values did not yield acceptable log-likelihood for any Kcd values (Figure 5E). Similar to analysis of metabolic demand variations, the best-fit Kcd values also correlated well with pre-PHx ultrasound measures. Liver volume (R = 0.53, *p* = 0.0039), and portal flow rate (R = −0.42, *p* = 0.031) both correlated with Kcd (Supplementary Figure S3).

**FIGURE 6:**
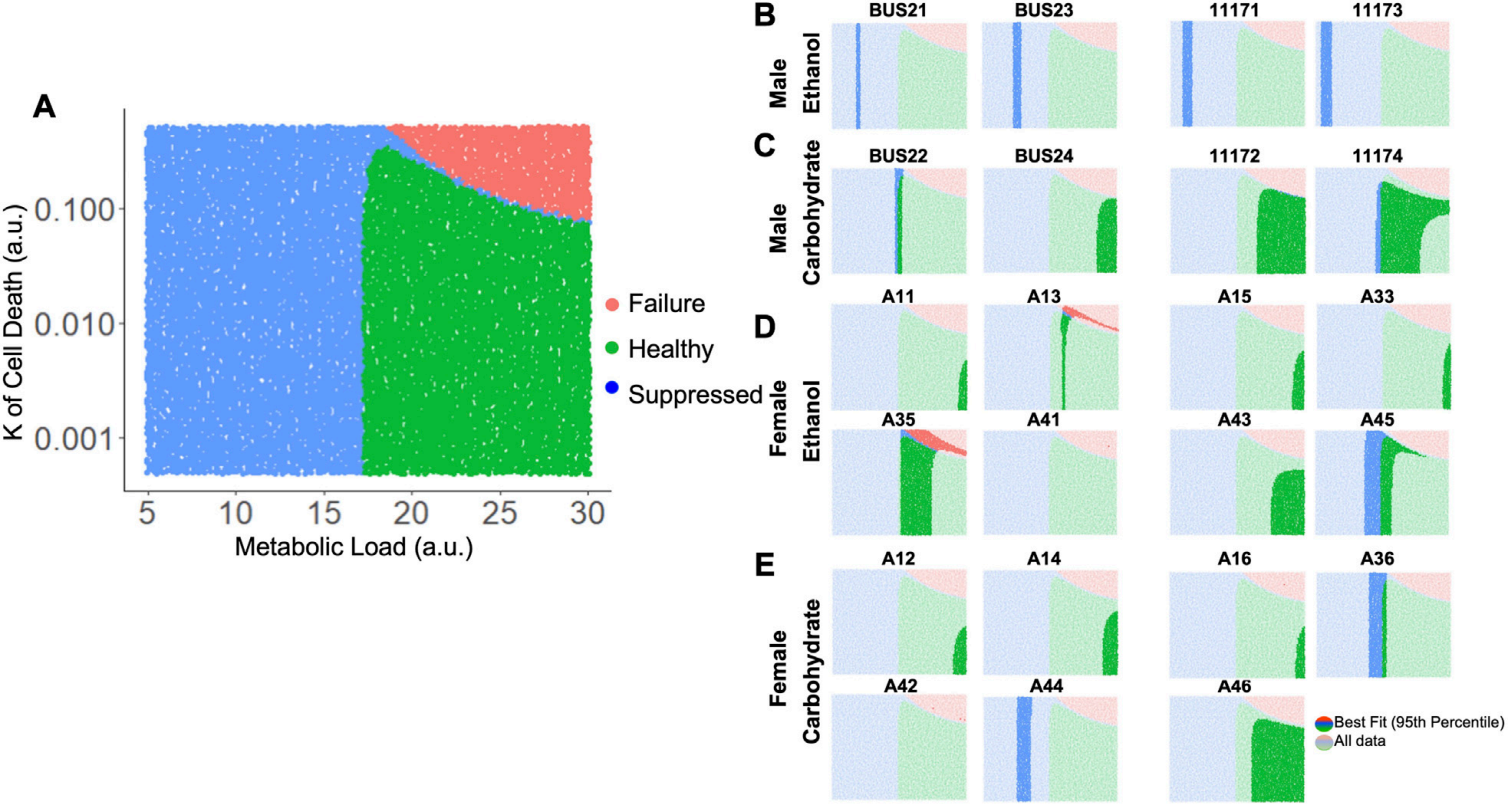
Matching model-predicted recovered liver volume of liver after 70% PHx to experimental data. **(A)** Categorized simulation results for variations in the parameters M and Kcd. Each dot represents a simulation for a specific M and Kcd values. The simulations were categorized based on final liver volume at 1-week post-PHx. For all panels, green indicates >85% of original volume (Healthy), blue indicates between 40% and 85% of original volume (Suppressed), and red indicates <40% of original volume (Failure). **(B–E)** show the same simulation categories as in A, highlighting the regions with best fits to experimentally observed liver volume time series data (95th percentile of log-likelihood) for individual ethanol- and carbohydrate-fed animals. Labels above each plot indicate unique animal identifiers.

Taking these results into account, we varied both M and Kcd in tandem, to explore the impact on the liver volume recovery for a range of combinatorial scenarios of metabolic demand and cell death sensitivity. Within this parametric landscape, we found regions that corresponded to high log-likelihood with experimental data. In most cases, increasing Kcd at the optimal M value eventually leads to a poor fit (Figures 5D, E). However, ethanol-fed males yielded a unique pattern in which a small range of M values (∼10–15) gave a good fit to the observed data with no dependence on Kcd within the given range (Figure 5C). This pattern seems to be due to the fact that at higher M values, increased Kcd leads to liver failure in the model simulations (Figure 6A). We categorized the model-predicted volume into healthy, suppressed and failure modes, similar to our previous approach (Verma et al., 2018; Verma et al., 2019). Mapping the parameter space corresponding to 95%ile log-likelihood of model-experiment match for each animal onto this landscape highlighted the differences in the ethanol-fed male group with all other experimental groups (Figures 6B–E; Supplementary Figure S4). The ethanol-fed males were unique in displaying best fit values only in the parameter space that corresponds to suppressed regeneration (<85% volume recovery at 1 week; Figure 6B), while other animals showed good fit of the simulations to experimental data in all the three modes of regeneration (Figures 6C–E). The apparent good fit of the failure modes to experimental data in two animals may have occurred due to relatively better fit at earlier than later time points, yielding high log-likelihood even though the model predicts reduction in liver volume at later time points. The ethanol-fed animals mapped more consistently to lower metabolic demand levels without significant impact of variations in the cell death sensitivity parameter, suggesting that the physiological stimulus of metabolic demand is critical in controlling liver regeneration in the case of chronic ethanol-fed male rats.

### Validation of end point physiology by immunohistochemistry

**FIGURE 7:**
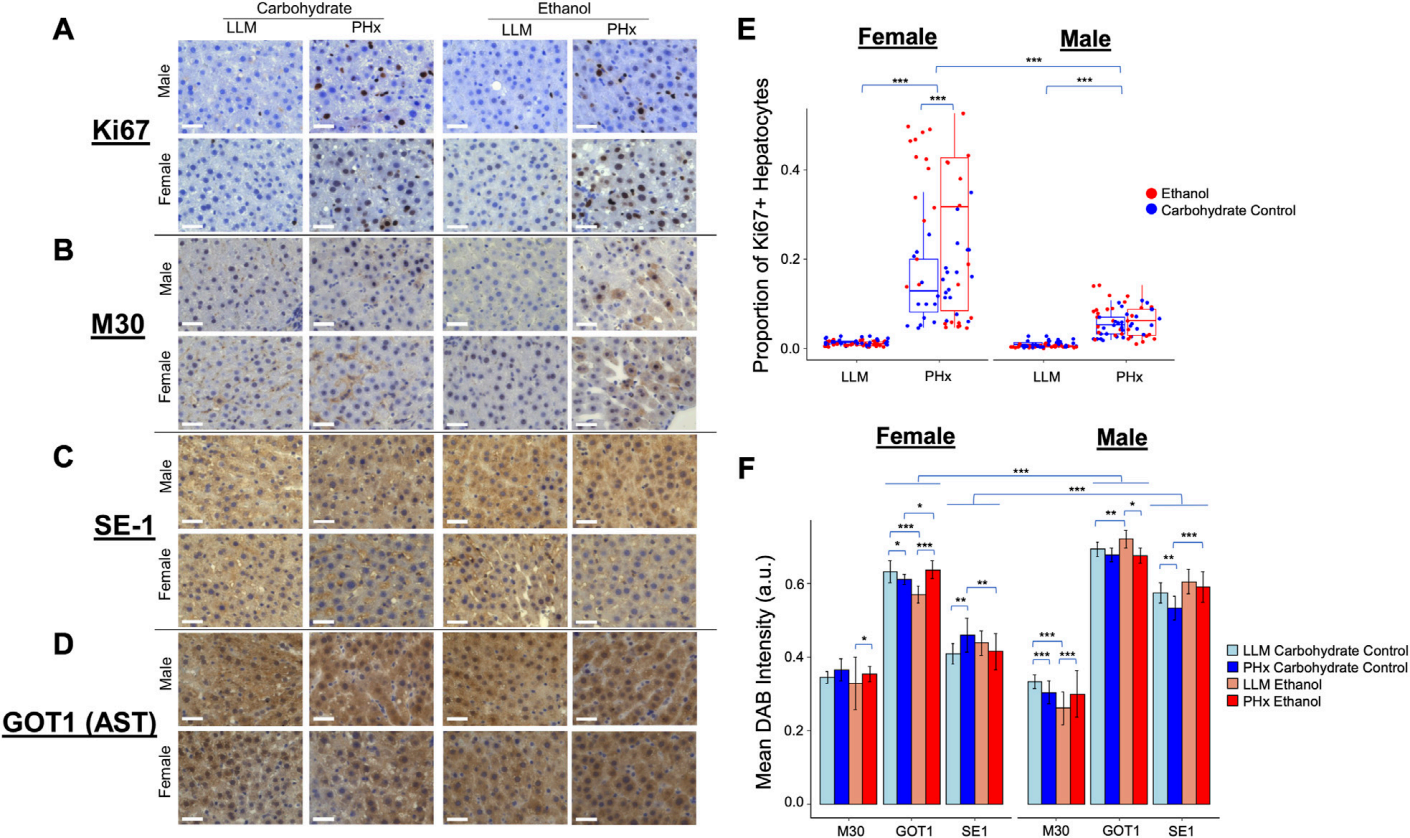
Immunohistochemisty validation of physiological dynamics. **(A–D)** Representative images taken with ×40 objective microscope lens. All images show DAB-Chromigen stain (brown) bound to primary antibody indicated on the left, with hematoxylin staining all nuclei (blue). Scale bar: 50 μm. **(E)** Proportion of hepatocytes from all Ki67-labeled images positive for DAB. Each point represents the average of ∼800 cells from a single 10X image. **(F)** Mean DAB Intensity for each primary antibody of interest shown on the x-axis. Error bars represent standard deviation. For all panels, *** indicates *p* < 0.001, ** indicates *p* < 0.01, and * indicates *p* < 0.05.

To connect the dynamics observed in the physiology and computational modeling simulations to relevant molecular changes in the liver, immunohistochemistry was performed using the resected liver tissue and liver tissue collected at 1-week post-PHx (LLM, t = 0; Figures 7A–D). The expression of Ki67 was used as a marker of cellular proliferation, and a marked increase was observed across all animals at 1-week post-PHx compared to pre-surgery levels. Female animals on average had a higher rate of proliferation than males at this later time point, with ethanol-fed females showing the highest rate (Figure 7E). This suggests that ethanol-fed female rats may have delayed cellular proliferation compared to the other experimental groups, though there was no delay seen in the volume regrowth (Figure 2A). Caspase-cleaved keratin-18 (M30) was used as a marker of liver-cell-specific cell death (Mueller et al., 2017). One week after PHx showed an upregulation of M30 in both male and female ethanol-fed animals, but a downregulation and no change in male and female controls, respectively (Figure 7F). When comparing male and female groups directly, females showed significantly higher cell death levels in response to both PHx and chronic-ethanol feeding (Supplementary Figures S5A, B). This corresponds to the results of parameter scanning in our computational model, where male ethanol-fed rats show a lack of sensitivity to the rate of cell death, and all female animals show data that fits limited cell death rates (Figure 5E; Supplementary Figures S5). The combined data could indicate cellular senescence induced by the combination of chronic-ethanol and PHx in males, while female animals maintain appropriate cell death responses.

To continue to emphasize the sex-based differences in the rat response to PHx and chronic ethanol, immunohistochemistry was performed on an additional marker that showed significant expression changes due to chronic ethanol and PHx in RNAseq data from rats of the same cohort (not shown). This marker was glutamic-oxaloacetic transaminase 1 (Got1), also known as aspartate aminotransferase 1, or AST. Immunohistochemistry showed significant upregulation in male rats across all samples (Figure 7F). Among the ethanol-fed animals, male and female rats showed opposite responses to PHx, where females upregulated Got1 expression at 1 week after PHx, males downregulated it significantly. Neither male nor female control-fed animals showed large changes in Got1 due to PHx (Supplementary Figures S5C, D). In this way, Got1 seems to demonstrate similar dynamics to that of sex-based differences in tissue oxygen saturation observed *via* ultrasound, where only ethanol-fed female rats had an upregulation at 1-week post-PHx.

Finally, a marker for hepatic sinusoidal endothelial cells was used to quantify changes in the liver sinusoids. The presence of SE-1 was more prevalent in male rats across all experimental groups, but the expression in all ethanol-fed animals was unchanged 1 week after PHx. Control-fed male rats showed a small downregulation in sinusoidal endothelial cells after PHx, while control-fed female rats showed a slight upregulation of the same marker after PHx (Figure 7F; Supplementary Figures S5E, F). We expected to see more SE-1-positive cells in the ethanol-fed female rats to account for the higher observed stiffness at 2-week post-PHx in those animals. In order to understand the disparity, we evaluated the proliferation of non-parenchymal cells using Ki67 as a immunohistochemical marker and quantitatively assessed the level of Ki67-positive non-parenchymal cells in all conditions (Supplementary Figure S6). Our results suggest that the observed disparity between SE-1 and stiffness may be explained by the increased number of Ki67-positive non-parenchymal cells see in the immunohistochemistry (Supplementary Figures S7).

## Discussion

Here, we present multi-method ultrasound data for 43 rats (23 male and 20 female) to study sex-dependent physiology in response to 70% partial hepatectomy and chronic ethanol intake. Our results align with previous studies of Sprague-Dawley rats treated with the Lieber-DeCarli liquid ethanol and control diets. While others have shown the ethanol diet decreases mass recovery after PHx (Diehl et al., 1990; Koniaris et al., 2003), our study is the first to demonstrate this phenomenon in a high-resolution, longitudinal, non-invasive assessment of *in vivo* liver volume as a function of time. Surprisingly, this ethanol-impaired volume recovery was not seen in female rats, despite previous evidence that female rodents are more susceptible to ethanol-mediated liver dysfunction and damage (Tanaka, et al., 1993; Fulham and Mandrekar, 2016). This raises questions about the mechanism wherein chronic ethanol intake is found to be a higher risk factor for liver disease in female individuals (Sato et al., 2001; Vatsalya et al., 2016).

Relating this unique dataset to a computational model of liver regeneration revealed important results. The dynamics of ethanol-mediated suppression of regeneration were best modeled by a lower metabolic load parameter, without any dependence on the cell death rate constant. While there was much variability in the dataset, all experimental groups (excepting male ethanol-fed) best fit a parameter space likely to result in healthy regeneration, which was dependent on both metabolic demand and cell death sensitivity in combination. This follows the phenomenon that cell death occurs as a response proportional to the mass of resected liver (Rehman et al., 2011), and is in line with the results of earlier simulations of the same computational model (Verma et al., 2018; Verma et al., 2019). With the assumption that cell death shapes the rate of healthy liver regeneration, we infer that chronic ethanol reduces the regenerative stimulus (e.g., metabolic load) to impair regeneration, with either a reduction or no change in cellular apoptosis induced by PHx.

Indeed, among the chronic-ethanol-fed rats in our study, we show lower presence of apoptosis marker M30 in males compared to females, both before and after PHx. We also show a lower rate of cellular proliferation as late as 1 week following PHx, as indicated by Ki67 expression in male rats compared to females, in line with the lower metabolic load predicted by the computational model. Further, these best-fit parameters were correlated with multiple pre-PHx non-invasive measures. Thus, it seems feasible that a similar computational model could be fit to human patient ultrasound volumetric data and provide predictive information about recovery from resection, in line with modeling using CT volumetric data (Verma et al., 2018; Verma et al., 2019). The technological enhancement in non-invasive imaging systems offers capabilities to collect physiological information across various organs and individuals. Doppler-based ultrasound methods are used to measure liver volumes in living donor liver transplantation (Hyun et al., 2017), and elastography approaches are used to measure target organ stiffness (Berzigotti, 2017). The widespread availability and portability of ultrasound-based non-invasive imaging systems offer the possibility of developing individualized computational models for patients with liver disease.

The present study also addresses questions related to changes in blood flow during partial hepatectomy response. This is of particular importance, as shown in a recent discovery relating the mechanical force of blood flow through the liver to growth factor signaling (Lorenz et al., 2018; Rabbany and Rafii, 2018). From a systematic perspective, we designed the present study to investigate how changes in blood flow after PHx are linked to the regenerative outcome. Given that portal hypertension is a common symptom of chronic liver injury (Berzigotti, 2017), we surmised that dysregulated blood flow may drive chronic liver injury and have implications for impaired regeneration in those pathologies. The study presented here suggests longitudinal hemodynamic data can capture pathological changes in blood flow to the liver, particularly in response to resection.

These results challenge the idea that partial hepatectomy concurrently induces increased blood flow from the portal vein and lowers flow from the hepatic artery in proportion to loss of tissue (Michalopoulos, 2010), thought to be caused by increased resistance in the vessel resulting from tissue loss (Audebert et al., 2017). The non-invasive quantification of volumetric flow rates shows increased flow per unit tissue through both vessels directly after PHx. However, this increased supply of oxygen-rich blood was highly variable in the rats in our experiments.

Interestingly, volumetric flow rates through the portal vein had the most negative correlation with liver volume recovery after 72 h, where we observed the largest differences between ethanol-fed males and control groups. It is important to note that blood flow rates measured here are not analogous to blood pressures, which were not collected in the present study. Additionally, animals were not fasted prior to data collection, despite evidence that feeding is able to effect rate of blood flow into the liver (Reininger and Spirstein, 1957). Yet, high portal pressure in human liver resection and transplant patients correlates with poor outcomes (Aguiar et al., 2011; Yang et al., 2016; Miyagi et al., 2018). Conversely, surgically implanted shunts that limit increase in portal pressure can severely impair the regrowth response (Marubashi et al., 2004). Our finding that rats with suppressed liver regeneration tend to show increased portal flow may be related to the evidence of the present study that the same animals have decreased metabolic load. For example, while mice exhibit significant decreases in blood glucose early in a PHx response (Gazit et al., 2010), rats maintain blood glucose levels after PHx (Paloheimo et al., 1984; Crumm et al., 2008). Increased portal flow likely provides nutrients that supplement the metabolic function of the liver to maintain glucose levels. Further work is needed to define proper hemodynamic regulation after liver resection, and its dysregulation during chronic liver injury.

A surprising finding of this study was the persistent stiffness induced by PHx in all animals. Shear wave elastography indicated significantly increased stiffness up to 72 h after PHx, when most animals had already recovered close to 80% of the original liver volume. These elevated stiffness measurements are likely to be affected by edema and hypertrophy of hepatocytes during regeneration. Previous studies have shown slight correlations between stiffness and portal pressure (Castera et al., 2012; Berzigotti, 2017; Elkrief et al., 2018). Hepatocyte size measurements in male rats after PHx show significant increase at 24 h post-PHx (Miyaoka et al., 2012). Importantly, stiffness measurements were below that which would be considered as fibrosis in rats (Hu et al., 2015; Hu et al., 2017), in line with the absence of fibrosis in previous histological studies of the alcohol-feeding model (Diehl, et al., 1990; Koniaris et al., 2003; Juskeviciute et al., 2016). However, we report increases in liver stiffness similar to what has been shown in a rat model of hepatic sinusoidal obstruction syndrome (Han et al., 2019), as well as human transplant donors who underwent a liver resection (Ninomiya et al., 2011). Additionally, extracellular matrix rebuilding could contribute to elevated stiffness, but only at later time points (72 h post-PHx) when this rebuilding is thought to occur (Michalopoulos, 2010; Juskeviciute et al., 2016).

Lastly, measures of oxygen saturation in the liver indicate that impaired regeneration after PHx in male ethanol-fed rats coincides with impaired recovery of oxygen saturation. While several reports show hypoxia has a positive effect on liver regeneration (Kron et al., 2016; Schadde et al., 2017), the results of this study support those in CCl4-treated rats, where chronic ethanol intake induces hypoxia in injured rat livers (Sato et al., 1983). Sato et al. cite decreased blood flow to the liver, dysfunction in microcirculation, and increased oxygen consumption by the liver to account for the decreased oxygen tension. Although the present study shows increased flow blood flow 1–10 h following PHx, the lowered oxygen levels are seen three or more days following PHx. At these time points, it is also unlikely that microcirculation would be significantly disrupted, as previous studies in this model show only steatosis in the way of histological abnormalities (Diehl, et al., 1990; Koniaris et al., 2003; Juskeviciute et al., 2016). Therefore, our results suggest increased oxygen consumption in male ethanol-fed rats after partial hepatectomy. It is likely that the changes in oxygen consumption may be reflected in the tissue ATP levels in the liver. A previous study has shown that ATP levels increase immediately within seconds in the venous circulation following PHx, presumably released from hepatocyte lysosomal compartments (Gonzales et al., 2010). An earlier study by one of the present co-authors reported that tissue levels of ATP are reduced immediately following PHx and remained at lower levels at least until 48 h (Crumm et al., 2008). These results are in contrast to our interpretation of lower oxygen levels as indicating increased oxygen consumption. A recent study on tumor tissue metabolism demonstrated that there are significant changes in both production and consumption of ATP in proliferating cells such that the ATP levels were rebalanced without a net change in the overall ATP levels (Bartman et al., 2023). It is possible that such a rebalancing of oxygen consumption, ATP production and biosynthetic processes may occur in the proliferating liver tissue post PHx.

In conclusion, the data here represent a reproducible protocol for non-invasive physiological assessment after both chronic ethanol feeding and 70% PHx. While raising many questions about the mechanisms that drive sex-dependent effects of ethanol on liver regeneration, computational modeling and molecular validation provide avenues to enhance understanding. Taking previous literature into account, the data shown here open new avenues for investigation into the importance of organ-scale regulation of blood flow into the liver at homeostasis and during regeneration.

## Data Availability

The original contributions presented in the study are included in the article/Supplementary Materials, further inquiries can be directed to the corresponding author.

## References

[B1] Aguiar L. R. F. D. Nassif P. A. N. Ribas C. A. P. M. Czeczko N. G. Ribas M. M. Marinho Júnior C. H. (2011). Regeneração do fígado após hepatectomia parcial em ratos submetidos à hipertensão portal pós-hepática. ABCD, Arq. Bras. Cir. Dig. 24, 144–151. 10.1590/S0102-67202011000200011

[B2] Audebert C. Bekheit M. Bucur P. Vibert E. Vignon-Clementel I. E. (2017). Partial hepatectomy hemodynamics changes: Experimental data explained by closed-loop lumped modeling. J. Biomech. 50, 202–208. 10.1016/j.jbiomech.2016.11.037 27890535

[B3] Bartman C. R. Weilandt D. R. Shen Y. Lee W. D. Han Y. TeSlaa T. (2023). Slow TCA flux and ATP production in primary solid tumours but not metastases. Nature 614, 349–357. 10.1038/s41586-022-05661-6 36725930 PMC10288502

[B4] Beatson R. Graham R. Grundland Freile F. Cozzetto D. Kannambath S. Pfeifer E. (2020). Cancer-associated hypersialylated MUC1 drives the differentiation of human monocytes into macrophages with a pathogenic phenotype. Commun. Biol. 3, 644. 10.1038/s42003-020-01359-5 33149188 PMC7642421

[B5] Berzigotti A. (2017). Non-invasive evaluation of portal hypertension using ultrasound elastography. J. Hepatol. 67, 399–411. 10.1016/j.jhep.2017.02.003 28223101

[B6] Brunt E. M. Janney C. G. Di Bisceglie A. M. Neuschwander-Tetri B. A. Bacon B. R. (1999). Nonalcoholic steatohepatitis: A proposal for grading and staging the histological lesions. Am. J. Gastroenterol. 94, 2467–2474. 10.1111/j.1572-0241.1999.01377.x 10484010

[B7] Castera L. Pinzani M. Bosch J. (2012). Non invasive evaluation of portal hypertension using transient elastography. J. Hepatol. 56, 696–703. 10.1016/j.jhep.2011.07.005 21767510

[B8] Choi S. H. Kwon J. H. Kim K. W. Jang H. Y. Kim J. H. Kwon H.-J. (2017). Measurement of liver volumes by portal vein flow by Doppler ultrasound in living donor liver transplantation. Clin. Transpl. 31, e13050. 10.1111/ctr.13050 28681460

[B9] Christ B. Dahmen U. Herrmann K.-H. König M. Reichenbach J. R. Ricken T. (2017). Computational modeling in liver surgery. Front. Physiol. 8, 906. 10.3389/fphys.2017.00906 29249974 PMC5715340

[B10] Clarke M. P. Kane R. A. Steele G. Hamilton E. S. Ravikumar T. S. Onik G. (1989). Prospective comparison of preoperative imaging and intraoperative ultrasonography in the detection of liver tumors. Surgery 106, 849–855. 2554519

[B11] Cook D. Ogunnaike B. A. Vadigepalli R. (2015). Systems analysis of non-parenchymal cell modulation of liver repair across multiple regeneration modes. BMC Syst. Biol. 9, 71. 10.1186/s12918-015-0220-9 26493454 PMC4618752

[B13] Crumm S. Cofan M. Juskeviciute E. Hoek J. B. (2008). Adenine nucleotide changes in the remnant liver: An early signal for regeneration after partial hepatectomy. Hepatology 48, 898–908. 10.1002/hep.22421 18697206 PMC3348855

[B14] de Graaf W. Bennink R. J. Heger M. Maas A. de Bruin K. van Gulik T. M. (2011). Quantitative assessment of hepatic function during liver regeneration in a standardized rat model. J. Nucl. Med. 52, 294–302. 10.2967/jnumed.110.078360 21233172

[B15] Diehl A. M. Thorgeirsson S. S. Steer C. J. (1990). Ethanol inhibits liver regeneration in rats without reducing transcripts of key protooncogenes. Gastroenterology 99, 1105–1112. 10.1016/0016-5085(90)90631-a 2394331

[B16] Dippold R. P. Vadigepalli R. Gonye G. E. Hoek J. B. (2012). Chronic ethanol feeding enhances miR-21 induction during liver regeneration while inhibiting proliferation in rats. Am. J. Physiol. Gastrointest. Liver Physiol. 303, G733–G743. 10.1152/ajpgi.00019.2012 22790595 PMC3468539

[B17] Duguay L. Coutu D. Hetu C. Joly J. G. (1982). Inhibition of liver regeneration by chronic alcohol administration. Gut 23, 8–13. 10.1136/gut.23.1.8 7056500 PMC1419599

[B18] Elkrief L. Ronot M. Andrade F. Dioguardi Burgio M. Issoufaly T. Zappa M. (2018). Non-invasive evaluation of portal hypertension using shear-wave elastography: Analysis of two algorithms combining liver and spleen stiffness in 191 patients with cirrhosis. Aliment. Pharmacol. Ther. 47, 621–630. 10.1111/apt.14488 29322599

[B19] Erdemir A. Mulugeta L. Ku J. P. Drach A. Horner M. Morrison T. M. (2020). Credible practice of modeling and simulation in healthcare: Ten rules from a multidisciplinary perspective. J. Transl. Med. 18, 369. 10.1186/s12967-020-02540-4 32993675 PMC7526418

[B20] Fulham M. A. Mandrekar P. (2016). Sexual dimorphism in alcohol induced adipose inflammation relates to liver injury. PLoS ONE 11, e0164225. 10.1371/journal.pone.0164225 27711160 PMC5053524

[B21] Gazit V. Weymann A. Hartman E. Finck B. N. Hruz P. W. Tzekov A. (2010). Liver regeneration is impaired in lipodystrophic fatty liver dystrophy mice. Hepatology 52, 2109–2117. 10.1002/hep.23920 20967828 PMC2991544

[B22] Gerling M. Zhao Y. Nania S. Norberg K. J. Verbeke C. S. Englert B. (2014). Real-time assessment of tissue hypoxia *in vivo* with combined photoacoustics and high-frequency ultrasound. Theranostics 4, 604–613. 10.7150/thno.7996 24723982 PMC3982131

[B23] Gonzales E. Julien B. Serrière-Lanneau V. Nicou A. Doignon I. Lagoudakis L. (2010). ATP release after partial hepatectomy regulates liver regeneration in the rat. J. Hepatol. 52, 54–62. 10.1016/j.jhep.2009.10.005 19914731 PMC3625734

[B24] Han H. Yang J. Li X. Zhuge Y.-Z. Zhu C.-K. Chen J. (2019). Role of virtual touch tissue imaging quantification in the assessment of hepatic sinusoidal obstruction syndrome in a rat model. J. Ultrasound Med. 38, 2039–2046. 10.1002/jum.14893 30561767

[B25] Hernández-Muñoz R. Lucinda Contreras-Zentella M. (2019). Involvement of cell oxidant status and redox state in the increased non-enzymatic ethanol oxidation by the regenerating rat liver. Biochem. Pharmacol. 161, 63–72. 10.1016/j.bcp.2019.01.003 30625299

[B26] Higgins G. M. Anderson R. M. (1931). Experimental pathology of the liver. “Restoration of the liver of the white rat following partial surgical removal. Arch. Pathol. 12, 186–202.

[B27] Hockings P. D. Roberts T. Campbell S. P. Reid D. G. Greenhill R. W. Polley S. R. (2002). Longitudinal magnetic resonance imaging quantitation of rat liver regeneration after partial hepatectomy. Toxicol. Pathol. 30, 606–610. 10.1080/01926230290105811 12371670

[B28] Hoskins P. R. (2011). Estimation of blood velocity, volumetric flow and wall shear rate using Doppler ultrasound. Ultrasound 19, 120–129. 10.1258/ult.2011.011015

[B29] Hu X.-D. Geng H.-Y. Wang L. Xu H.-F. Su Y. Liang S. (2017). Supersonic shear wave imaging of the spleen for staging of liver fibrosis in rats. Ultrasound Med. Biol. 43, 2343–2351. 10.1016/j.ultrasmedbio.2017.04.013 28705556

[B30] Hu Z. Luo J. Wei H. Ou W. Xiao S. Gan M. (2015). Correlation of virtual touch tissue quantification and liver biopsy in a rat liver fibrosis model. Mol. Med. Rep. 11, 3694–3700. 10.3892/mmr.2015.3209 25592825

[B31] Hysi E. He X. Fadhel M. N. Zhang T. Krizova A. Ordon M. (2020). Photoacoustic imaging of kidney fibrosis for assessing pretransplant organ quality. JCI Insight 5, e136995. 10.1172/jci.insight.136995 32298239 PMC7259526

[B32] Jayakumar S. Middleton M. S. Lawitz E. J. Mantry P. S. Caldwell S. H. Arnold H. (2019). Longitudinal correlations between MRE, MRI-PDFF, and liver histology in patients with non-alcoholic steatohepatitis: Analysis of data from a phase II trial of selonsertib. J. Hepatol. 70, 133–141. 10.1016/j.jhep.2018.09.024 30291868

[B33] Juskeviciute E. Dippold R. P. Antony A. N. Swarup A. Vadigepalli R. Hoek J. B. (2016). Inhibition of miR-21 rescues liver regeneration after partial hepatectomy in ethanol-fed rats. Am. J. Physiol. Gastrointest. Liver Physiol. 311, G794–G806. 10.1152/ajpgi.00292.2016 27634014 PMC5130549

[B34] Karmacharya M. B. Sultan L. R. Kirkham B. M. Brice A. K. Wood A. K. W. Sehgal C. M. (2020). Photoacoustic imaging for assessing tissue oxygenation changes in rat hepatic fibrosis. Diagn. (Basel) 10, 705. 10.3390/diagnostics10090705 PMC7555416 32957666

[B35] Kassambara A. (2021). rstatix: Pipe-Friendly framework for basic statistical tests. R package version 0.7.0. Available at: https://CRAN.R-project.org/package=rstatix .

[B36] Koniaris L. G. McKillop I. H. Schwartz S. I. Zimmers T. A. (2003). Liver regeneration. J. Am. Coll. Surg. 197, 634–659. 10.1016/S1072-7515(03)00374-0 14522336

[B37] Kono H. Wheeler M. D. Rusyn I. Lin M. Seabra V. Rivera C. A. (2000). Gender differences in early alcohol-induced liver injury: Role of CD14, NF-kappaB, and TNF-alpha. Am. J. Physiol. Gastrointest. Liver Physiol. 278, G652–G661. 10.1152/ajpgi.2000.278.4.G652 10762620

[B38] Kron P. Linecker M. Limani P. Schlegel A. Kambakamba P. Lehn J.-M. (2016). Hypoxia-driven Hif2a coordinates mouse liver regeneration by coupling parenchymal growth to vascular expansion. Hepatology 64, 2198–2209. 10.1002/hep.28809 27628483

[B39] Liu C.-N. Morin J. Dokmanovich M. Bluette C. T. Goldstein R. Manickam B. (2019). Nanoparticle contrast-enhanced micro-CT: A preclinical tool for the 3D imaging of liver and spleen in longitudinal mouse studies. J. Pharmacol. Toxicol. Methods 96, 67–77. 10.1016/j.vascn.2019.02.003 30738209

[B40] Lorenz L. Axnick J. Buschmann T. Henning C. Urner S. Fang S. (2018). Mechanosensing by β1 integrin induces angiocrine signals for liver growth and survival. Nature 562, 128–132. 10.1038/s41586-018-0522-3 30258227

[B41] Mainenti P. P. Romano F. Pizzuti L. Segreto S. Storto G. Mannelli L. (2015). Non-invasive diagnostic imaging of colorectal liver metastases. World J. Radiol. 7, 157–169. 10.4329/wjr.v7.i7.157 26217455 PMC4506934

[B42] Marubashi S. Sakon M. Nagano H. Gotoh K. Hashimoto K. Kubota M. (2004). Effect of portal hemodynamics on liver regeneration studied in a novel portohepatic shunt rat model. Surgery 136, 1028–1037. 10.1016/j.surg.2004.03.012 15523397

[B43] Meier Y. Cavallaro M. Roos M. Pauli-Magnus C. Folkers G. Meier P. J. (2005). Incidence of drug-induced liver injury in medical inpatients. Eur. J. Clin. Pharmacol. 61, 135–143. 10.1007/s00228-004-0888-z 15726344

[B44] Michalopoulos G. K. Bhushan B. (2021). Liver regeneration: Biological and pathological mechanisms and implications. Nat. Rev. Gastroenterol. Hepatol. 18, 40–55. 10.1038/s41575-020-0342-4 32764740

[B45] Michalopoulos G. K. (2017). Hepatostat: Liver regeneration and normal liver tissue maintenance. Hepatology 65, 1384–1392. 10.1002/hep.28988 27997988

[B46] Michalopoulos G. K. (2010). Liver regeneration after partial hepatectomy: Critical analysis of mechanistic dilemmas. Am. J. Pathol. 176, 2–13. 10.2353/ajpath.2010.090675 20019184 PMC2797862

[B47] Miller C. M. Quintini C. Dhawan A. Durand F. Heimbach J. K. Kim-Schluger H. L. (2017). The international liver transplantation society living donor liver transplant recipient guideline. Transplantation 101, 938–944. 10.1097/TP.0000000000001571 28437386 PMC5642345

[B48] Miyagi S. Nakanishi C. Hara Y. Nakanishi W. Tokodai K. Shimizu K. (2018). Correlation between splenectomy and portal vein complications in living donor liver transplantation. Transpl. Proc. 50, 2611–2613. 10.1016/j.transproceed.2018.03.104 30401361

[B49] Miyaoka Y. Ebato K. Kato H. Arakawa S. Shimizu S. Miyajima A. (2012). Hypertrophy and unconventional cell division of hepatocytes underlie liver regeneration. Curr. Biol. 22, 1166–1175. 10.1016/j.cub.2012.05.016 22658593

[B50] Morales-González J. A. Gutiérrez-Salinas J. Hernández-Muñoz R. (1998). Pharmacokinetics of the ethanol bioavailability in the regenerating rat liver induced by partial hepatectomy. Alcohol. Clin. Exp. Res. 22, 1557–1563. 10.1111/j.1530-0277.1998.tb03949.x 9802542

[B51] Mueller S. Nahon P. Rausch V. Peccerella T. Silva I. Yagmur E. (2017). Caspase-cleaved keratin-18 fragments increase during alcohol withdrawal and predict liver-related death in patients with alcoholic liver disease. Hepatology 66, 96–107. 10.1002/hep.29099 28170108

[B52] Ninomiya M. Shirabe K. Ijichi H. Toshima T. Harada N. Uchiyama H. (2011). Temporal changes in the stiffness of the remnant liver and spleen after donor hepatectomy as assessed by acoustic radiation force impulse: A preliminary study. Hepatol. Res. 41, 579–586. 10.1111/j.1872-034X.2011.00809.x 21561532

[B53] Noureddin M. Lam J. Peterson M. R. Middleton M. Hamilton G. Le T.-A. (2013). Utility of magnetic resonance imaging versus histology for quantifying changes in liver fat in nonalcoholic fatty liver disease trials. Hepatology 58, 1930–1940. 10.1002/hep.26455 23696515 PMC4819962

[B54] Paloheimo M. Linkola J. Lempinen M. Folke M. (1984). Time-courses of hepatocellular hyperpolarization and cyclic adenosine 3′,5′-monophosphate accumulation after partial hepatectomy in the rat. Gastroenterology 87, 639–646. 10.1016/0016-5085(84)90538-9 6086441

[B55] Periwal V. Gaillard J. R. Needleman L. Doria C. (2014). Mathematical model of liver regeneration in human live donors. J. Cell. Physiol. 229, 599–606. 10.1002/jcp.24482 24446196 PMC6289189

[B56] R Core Team (2020). R: A language and environment for statistical computing. Vienna, Austria: R Foundation for Statistical Computing. Available at: https://www.R-project.org/ .

[B57] Rabbany S. Y. Rafii S. (2018). Blood flow forces liver growth. Nature 562, 42–43. 10.1038/d41586-018-06741-2 30275551

[B58] Rehman H. Sun J. Shi Y. Ramshesh V. K. Liu Q. Currin R. T. (2011). NIM811 prevents mitochondrial dysfunction, attenuates liver injury, and stimulates liver regeneration after massive hepatectomy. Transplantation 91, 406–412. 10.1097/TP.0b013e318204bdb2 21131897 PMC3399729

[B59] Reininger E. J. Sapirstein L. A. (1957). Effect of digestion on distribution of blood flow in the rat. Science 126, 1176. 10.1126/science.126.3284.1176 13495444

[B60] Sato N. Kamada T. Kawano S. Hayashi N. Kishida Y. Meren H. (1983). Effect of acute and chronic ethanol consumption on hepatic tissue oxygen tension in rats. Pharmacol. Biochem. Behav. 18 (1), 443–447. 10.1016/0091-3057(83)90215-0 6685303

[B61] Sato N. Lindros K. O. Baraona E. Ikejima K. Mezey E. Järveläinen H. A. (2001). Sex difference in alcohol-related organ injury. Alcohol. Clin. Exp. Res. 25, 40S–45S. 10.1097/00000374-200105051-00007 11391047

[B62] Schadde E. Tsatsaris C. Swiderska-Syn M. Breitenstein S. Urner M. Schimmer R. (2017). Hypoxia of the growing liver accelerates regeneration. Surgery 161, 666–679. 10.1016/j.surg.2016.05.018 27436690

[B63] Stirling D. R. Carpenter A. E. Cimini B. A. (2021a). CellProfiler Analyst 3.0: Accessible data exploration and machine learning for image analysis. Bioinformatics 37, 3992–3994. 10.1093/bioinformatics/btab634 PMC10186093 34478488

[B64] Stirling D. R. Swain-Bowden M. J. Lucas A. M. Carpenter A. E. Cimini B. A. Goodman A. (2021b). CellProfiler 4: Improvements in speed, utility and usability. BMC Bioinforma. 22, 433. 10.1186/s12859-021-04344-9 PMC8431850 34507520

[B65] Tanaka T. Kurai K. Kunitoh S. Kondo K. Goto Y. Kawai S. (1993). Gender-related differences in the inhibitory effect on liver regeneration in alcohol-treated rats: Study of polyamine metabolism. Alcohol Alcohol 1A, 15–20. Suppl. 1A. 10.1093/alcalc/28.supplement_1a.15 8141919

[B66] Taub R. (1996). Liver regeneration in health and disease. Clin. Lab. Med. 16, 341–360. 10.1016/s0272-2712(18)30273-7 8792076

[B67] Vatsalya V. Liaquat H. B. Ghosh K. Mokshagundam S. P. McClain C. J. (2016). A review on the sex differences in organ and system pathology with alcohol drinking. Curr. Drug Abuse Rev. 9, 87–92. 10.2174/1874473710666170125151410 28124600 PMC5894513

[B68] Verma B. K. Subramaniam P. Vadigepalli R. (2019). Model-based virtual patient analysis of human liver regeneration predicts critical perioperative factors controlling the dynamic mode of response to resection. BMC Syst. Biol. 13, 9. 10.1186/s12918-019-0678-y 30651095 PMC6335689

[B69] Verma B. K. Subramaniam P. Vadigepalli R. (2018). Modeling the dynamics of human liver failure post liver resection. Process. (Basel) 6, 115. 10.3390/pr6080115 PMC6534166 31131255

[B70] Wei H. Song B. (2020). Elastography for longitudinal assessment of liver fibrosis after antiviral therapy: A review. J. Clin. Transl. Hepatol. 8, 445–453. 10.14218/JCTH.2020.00033 33447528 PMC7782123

[B71] Will O. M. Damm T. Campbell G. M. von Schönfells W. Açil Y. Will M. (2017). Longitudinal micro-computed tomography monitoring of progressive liver regeneration in a mouse model of partial hepatectomy. Lab. Anim. 51, 422–426. 10.1177/0023677216678824 27932685

[B72] Yamamoto K. N. Ishii M. Inoue Y. Hirokawa F. MacArthur B. D. Nakamura A. (2016). Prediction of postoperative liver regeneration from clinical information using a data-led mathematical model. Sci. Rep. 6, 34214. 10.1038/srep34214 27694914 PMC5046126

[B73] Yang L. Luo Y. Ma L. Wang H. Ling W. Li J. (2016). Establishment of a novel rat model of different degrees of portal vein stenosis following 70% partial hepatectomy. Exp. Anim. 65, 165–173. 10.1538/expanim.15-0108 26822935 PMC4873485

[B74] Yu S. J. (2016). A concise review of updated guidelines regarding the management of hepatocellular carcinoma around the world: 2010-2016. Clin. Mol. Hepatol. 22, 7–17. 10.3350/cmh.2016.22.1.7 27044761 PMC4825164

[B75] Zafarnia S. Mrugalla A. Rix A. Doleschel D. Gremse F. Wolf S. D. (2019). Non-invasive imaging and modeling of liver regeneration after partial hepatectomy. Front. Physiol. 10, 904. 10.3389/fphys.2019.00904 31379606 PMC6652107

