# Supplementary figures and images for: Longitudinal ultrasound imaging and network modeling in rats reveal sex-dependent suppression of liver regeneration after resection in alcoholic liver disease
Source: Front Physiol. 2023 Mar 9;14:1102393. doi: 10.3389/fphys.2023.1102393 (PMC10033530; doi:10.3389/fphys.2023.1102393)

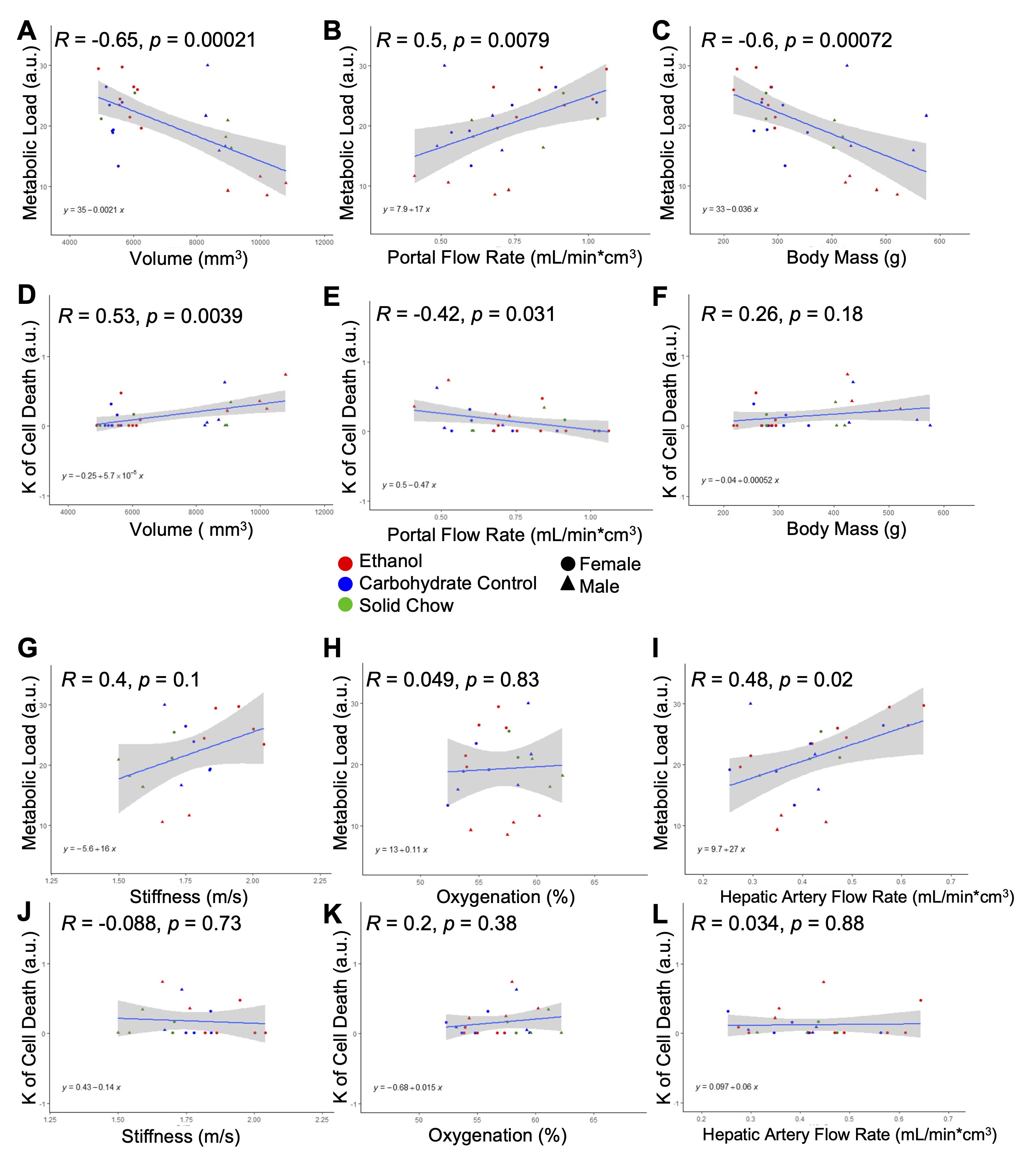

Supplement: Supplementary file 1 [file Image3.JPEG]

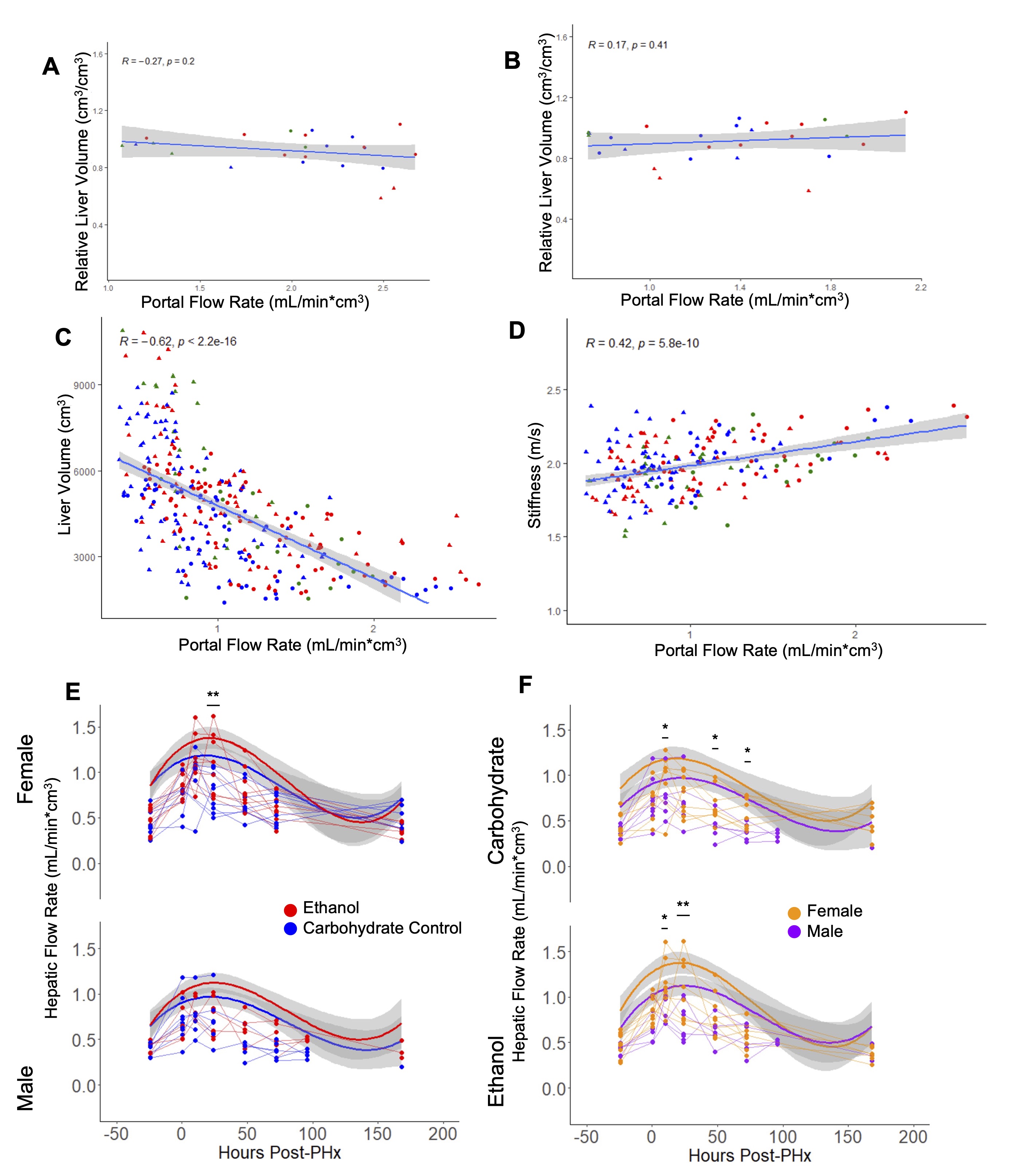

Supplement: Supplementary file 6 [file Image1.JPEG]

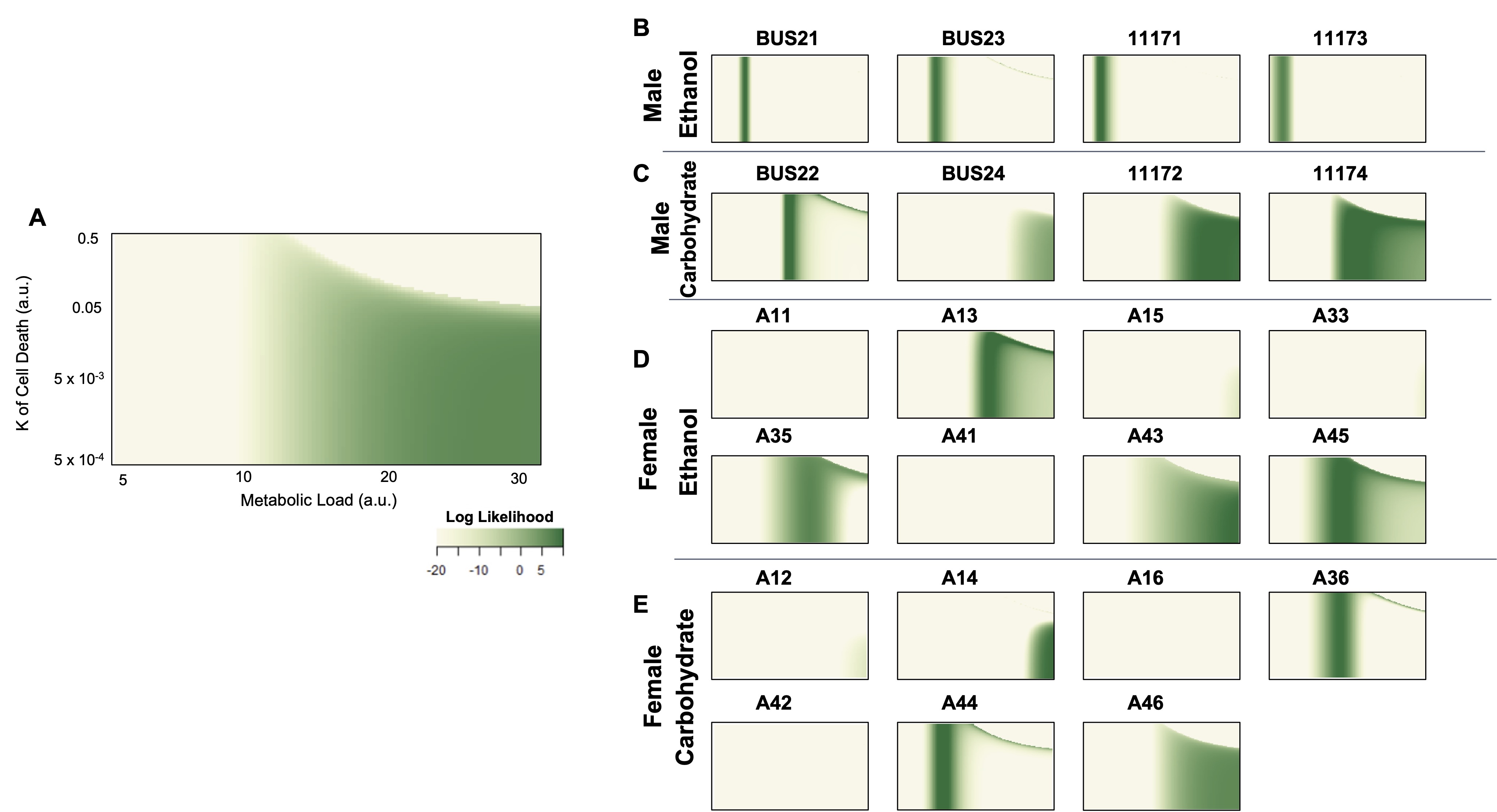

Supplement: Supplementary file 7 [file Image4.JPEG]

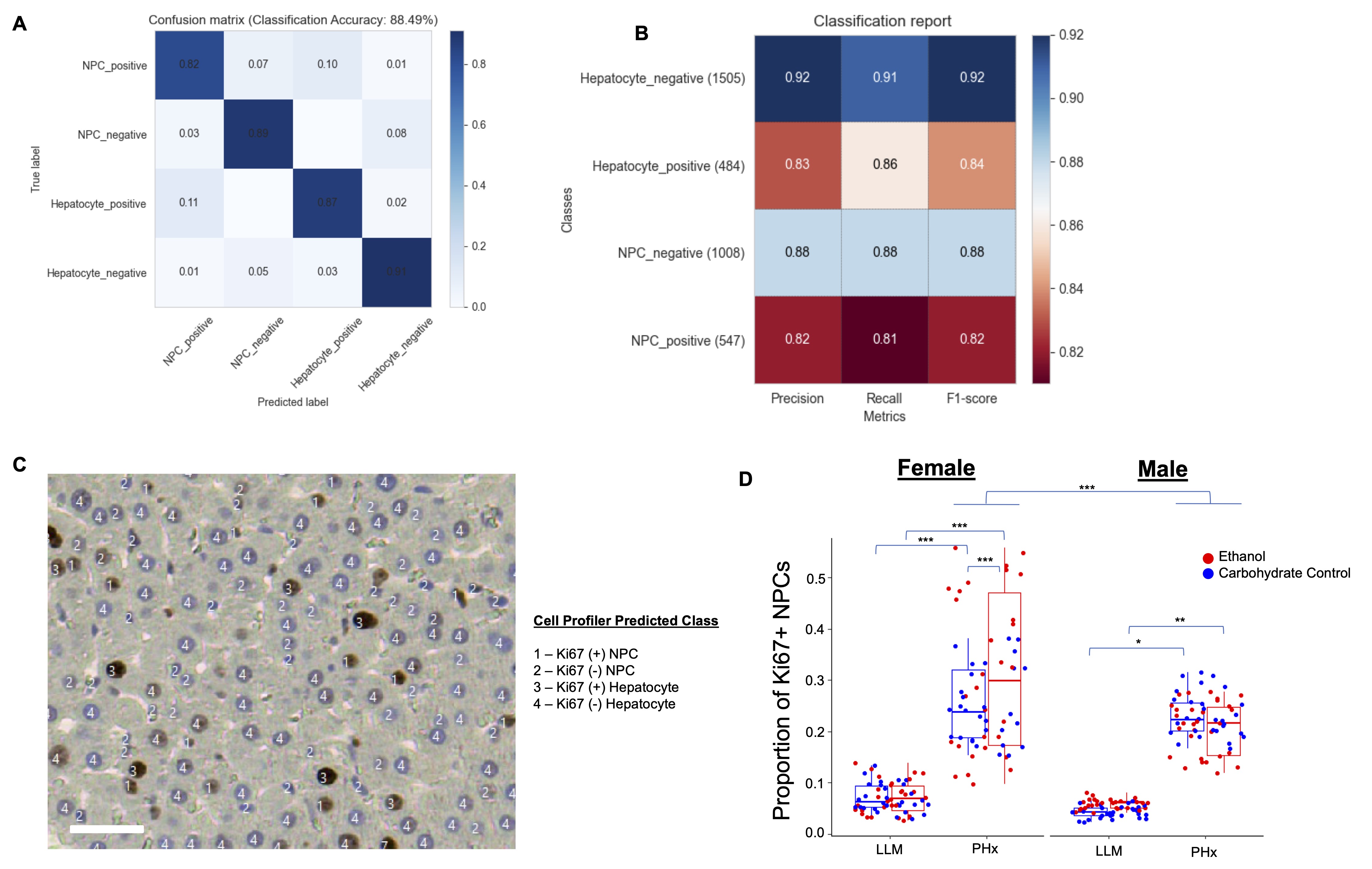

Supplement: Supplementary file 8 [file Image7.JPEG]

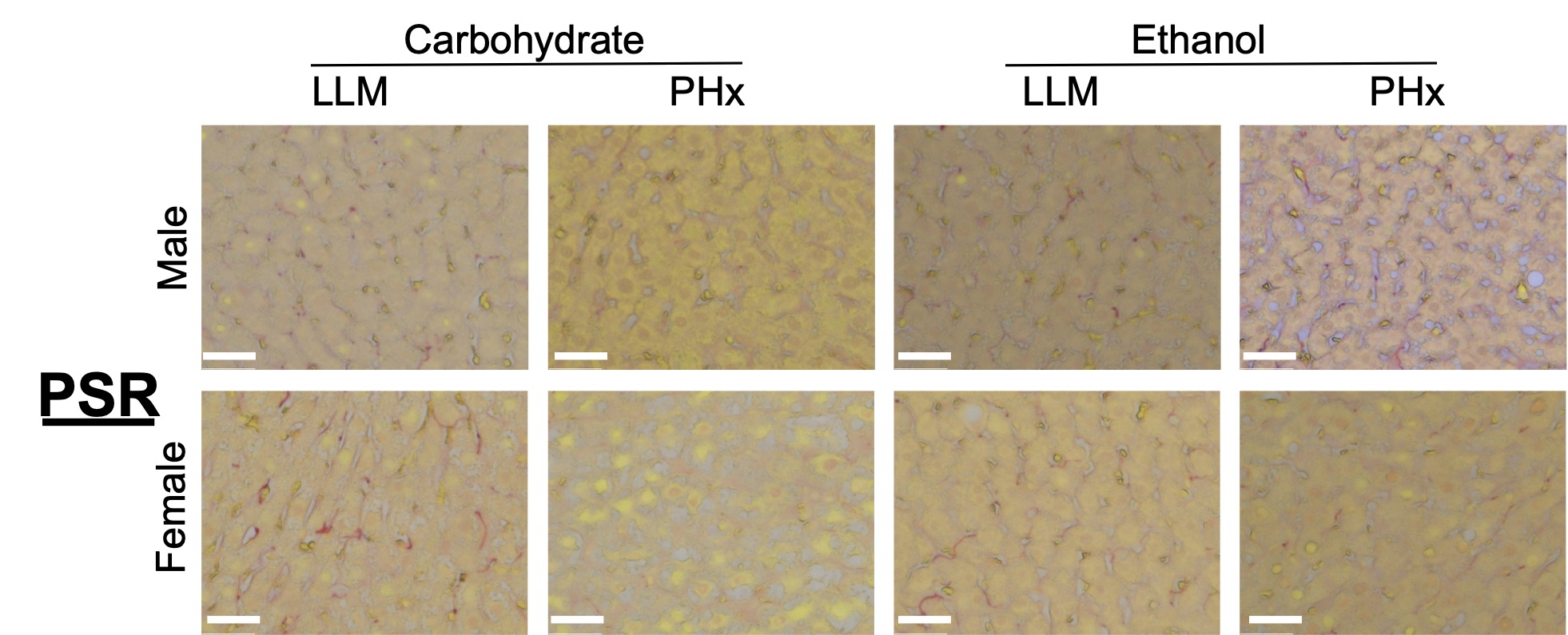

Supplement: Supplementary file 9 [file Image2.JPEG]

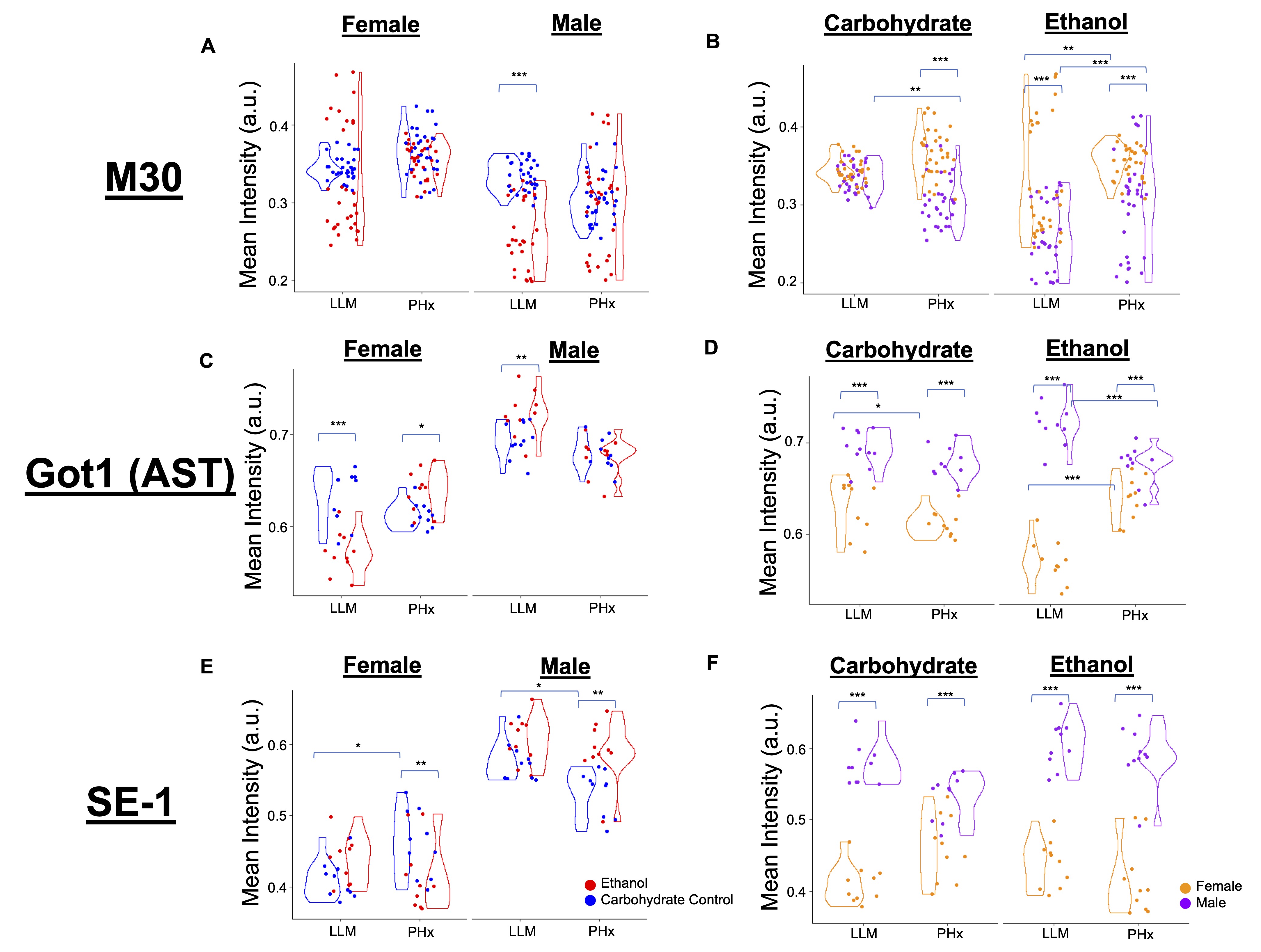

Supplement: Supplementary file 10 [file Image5.JPEG]

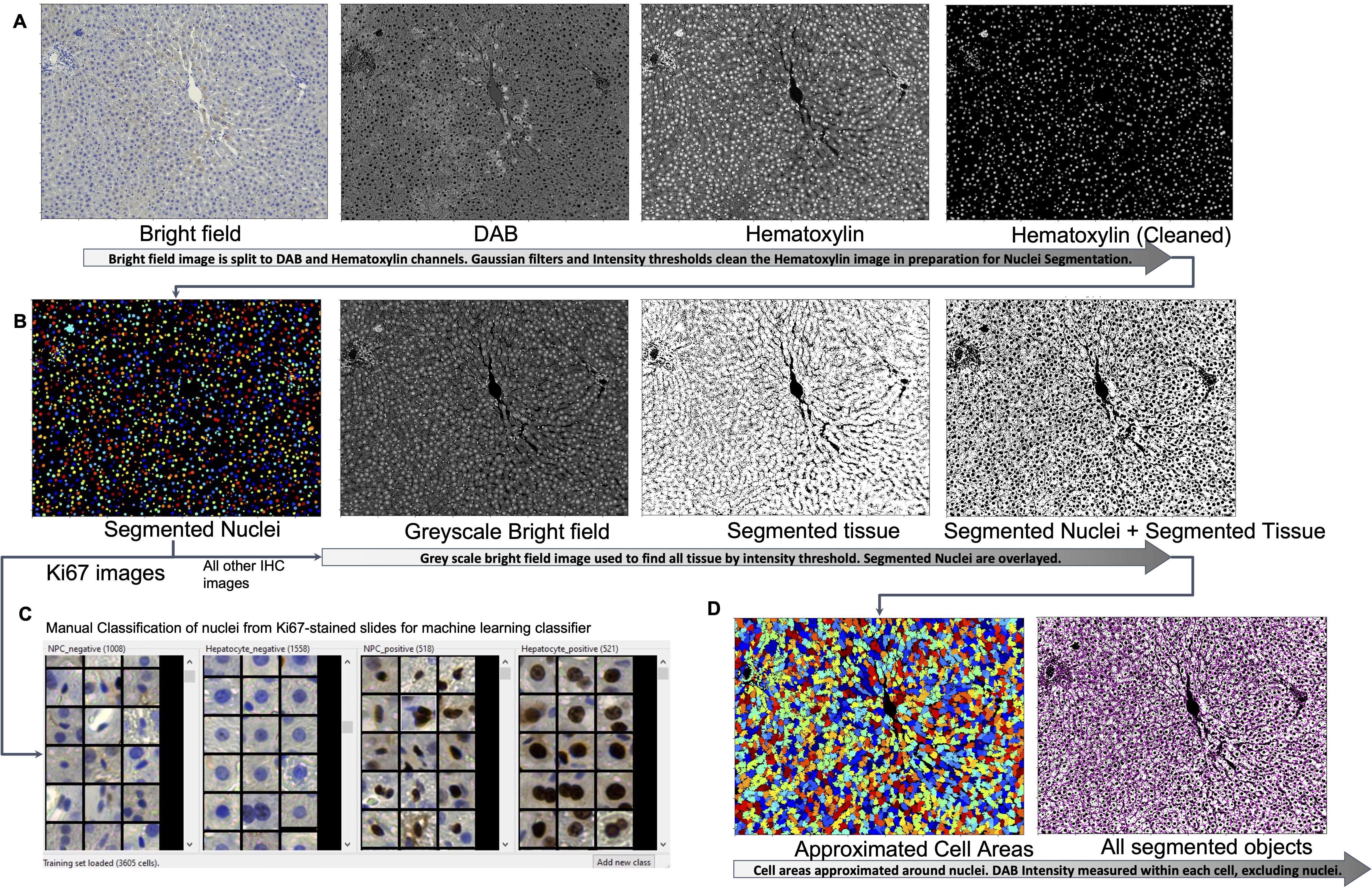

Supplement: Supplementary file 19 [file Image6.JPEG]
