# Supplementary material for: Longitudinal ultrasound imaging and network modeling in rats reveal sex-dependent suppression of liver regeneration after resection in alcoholic liver disease
Source: Front Physiol. 2023 Mar 9;14:1102393. doi: 10.3389/fphys.2023.1102393 (PMC10033530; doi:10.3389/fphys.2023.1102393)
Supplement: Supplementary file 4 [file Table1.docx]

**Supplementary Table 1:** List of parameters and values used in the computational model, adopted from Cook et al. (2015).

| **Parameter** | **Value** | **Scanned?** | **Description** |
| --- | --- | --- | --- |
| $M$ | 20.8 | Yes (linear sequence of range 5 - 30, and length 100) | Mechanical stress or increased nutrient and detoxification demand |
| $k_{IL6}$ | 1.5 | No | Rate of production of IL6 from non-parenchymal cells |
| $\kappa_{IL6}$ | 0.9 | No | Rate of IL6 degradation |
| $V_{JAK}$ | 20,000 | No | Maximum rate of activation of JAK |
| $K_{M}^{JAK}$ | 10,000 | No | Concentration of JAK, when the rate of activation of JAK is half of the maximum rate |
| $\kappa_{JAK}$ | 0.4 | No | Rate of degradation of JAK |
| $[proSTAT3]$ | 2 | No | Relative concentration of monomeric STAT3 |
| $V_{ST3}$ | 7500 | No | Maximum rate of STAT3 phosphorylation |
| $K_{M}^{ST3}$ | 0.4 | No | Michaelis-Menten concentration of proSTAT3 |
| $\kappa_{ST3}$ | 0.1 | No | Rate of dephosphorylation of proSTAT3 |
| $V_{SOCS3}$ | 24,000 | No | Maximum rate of SOCS3 activation |
| $K_{M}^{SOCS3}$ | 0.0007 | No | Concentration of SOCS3, when it’s rate of activation is half the maximum rate |
| $\kappa_{SOCS3}$ | 0.4 | No | Rate of degradation of SOCS3 |
| $K_{I}^{SOCS3}$ | 0.015 | No | SOCS3 inhibition constant on STAT3 |
| $V_{IE}$ | 250 | No | Maximum rate of activation of IE gene |
| $K_{M}^{IE}$ | 18 | No | Concentration of IE gene, when it’s rate of activation is half the maximum rate |
| $\kappa_{IE}$ | 5 | No | Rate of degradation of IE gene |
| $k_{deg}$ | 7 | No | Rate of degradation of ECM by MMPs |
| $\kappa_{ECM}$ | 33 | No | Rate of degradation of ECM |
| $k_{GF}$ | 0.113 | No | Rate of production of growth factor from non-parenchymal cells |
| $\kappa_{GF}$ | 0.23 | No | Rate of degradation of growth factor |
| $k_{up}$ | 0.06 | No | Rate of binding of growth factor to ECM |
| $k_{QP}$ | 0.007 | No | Rate of hepatocytes transition from quiescence to primed state |
| $k_{PR}$ | 0.0044 | No | Rate of hepatocytes transition from primed to replicating state |
| $k_{RQ}$ | 0.054 | No | Rate of hepatocytes transition from replicating to quiescence state |
| $k_{prol}$ | 0.02 | No | Rate of proliferation of hepatocytes |
| $k_{req}$ | 0.1 | No | Rate of re-quiescence of primed hepatocytes |
| $\theta_{req}$ | 8 | No | Re-quiescence parameter in the sigmoidal function (σ_req_) defining the threshold of re-quiescence |
| $\beta_{req}$ | 3 | No | Re-quiescence parameter in the sigmoidal function (σ_req_) defining the threshold of re-quiescence |
| $k_{cd}$ | 0.1 | Yes (logarithmic sequence of range 0.0005 - 0.5, and length 100) | Cell death rate of damaged hepatocytes |
| $\theta_{cd}$ | 0.009 | No | Cell death sensitivity parameter of the sigmoidal function (σ_cd_) |
| $\beta_{cd}$ | 0.0045 | No | Cell death sensitivity parameter of the sigmoidal function (σ_cd_) defining the cell death threshold |
| $k_{G}$ | 0.00035 | No | Rate of growth of relative cell mass |

**References:**

Cook, Daniel, Babatunde A. Ogunnaike, and Rajanikanth Vadigepalli. "Systems analysis of non-parenchymal cell modulation of liver repair across multiple regeneration modes." *BMC systems biology* 9, no. 1 (2015): 1-24.
