# Supplementary material for: Longitudinal ultrasound imaging and network modeling in rats reveal sex-dependent suppression of liver regeneration after resection in alcoholic liver disease
Source: Front Physiol. 2023 Mar 9;14:1102393. doi: 10.3389/fphys.2023.1102393 (PMC10033530; doi:10.3389/fphys.2023.1102393)
Supplement: Supplementary file 12 [file Table2.docx]

**Supplementary Table 2**. Fibrosis scores in the liver tissue samples generated using Visiopharm image analysis software and independently by a pathologist. LLM – left lateral and medial lobe liver tissue excised at partial hepatectomy surgery. PHx – liver tissue from one week after partial hepatectomy.

| **Animal** | **Sex** | **Diet** | **Tissue** | **Visiopharm Image Analysis** | | | | **Pathologist Scoring** |
| --- | --- | --- | --- | --- | --- | --- | --- | --- |
|  |  |  |  | Bridging fibrosis (%) | Fibrosis in periportal space (%) | Fibrosis in perisinusoidal space (%) | Fibrosis stage (Brunt) | Fibrosis score |
| A11057 | Male | Ethanol | LLM | 0.01 | 0.55 | 0.14 | 0 | F0 |
| A11057 | Male | Ethanol | PHx | 0.02 | 4.09 | 0.08 | 0 | F0 |
| A11058 | Male | Carbohydrate | LLM | 0.17 | 6.72 | 0.33 | 0 | F0 |
| A11058 | Male | Carbohydrate | PHx | 0.02 | 0.53 | 0.23 | 0 | F0 |
| A11111 | Male | Ethanol | LLM | 0.01 | 0.49 | 0.12 | 0 | F0 |
| A11111 | Male | Ethanol | PHx | 0 | 0.34 | 0.05 | 0 | F0 |
| A11112 | Male | Carbohydrate | LLM | 0.04 | 0.69 | 0.34 | 0 | F0 |
| A11112 | Male | Carbohydrate | PHx | 0.02 | 0.33 | 0.07 | 0 | F0 |
| A11 | Female | Ethanol | LLM | 0 | 0.38 | 0.09 | 0 | F0 |
| A11 | Female | Ethanol | PHx | 0 | 0.11 | 0.01 | 0 | F0 |
| A12 | Female | Carbohydrate | LLM | 0 | 0.34 | 0.13 | 0 | F0 |
| A12 | Female | Carbohydrate | PHx | 0 | 0.08 | 0.01 | 0 | F0 |
| A13 | Female | Ethanol | LLM | 0 | 0.44 | 0.13 | 0 | F0 |
| A13 | Female | Ethanol | PHx | 0 | 0.15 | 0.01 | 0 | F0 |
| A14 | Female | Carbohydrate | LLM | 0.05 | 0.49 | 0.26 | 0 | F0 |
| A14 | Female | Carbohydrate | PHx | 0 | 0.96 | 0.02 | 0 | F0 |
| A15 | Female | Ethanol | LLM | 0.03 | 0.51 | 0.18 | 0 | F0 |
| A15 | Female | Ethanol | PHx | 0 | 0.11 | 0.01 | 0 | F0 |
| A16 | Female | Carbohydrate | LLM | 0.01 | 0.4 | 0.12 | 0 | F0 |
| A16 | Female | Carbohydrate | PHx | 0 | 0.26 | 0.01 | 0 | F0 |
