# Supplementary material for: Longitudinal ultrasound imaging and network modeling in rats reveal sex-dependent suppression of liver regeneration after resection in alcoholic liver disease
Source: Front Physiol. 2023 Mar 9;14:1102393. doi: 10.3389/fphys.2023.1102393 (PMC10033530; doi:10.3389/fphys.2023.1102393)
Supplement: Supplementary file 14 [file DataSheet2.docx]

**Self-assessment of conformance to the Ten Simple Rules of Credible Practice in Modeling and Simulation in Healthcare**

Longitudinal ultrasound imaging and computational modeling reveal sex-dependent suppression of liver regeneration after resection in alcoholic liver disease

The following self-assessment is based on the rules specified in Erdemir et al. (2020) and the rubric available at: <https://www.imagwiki.nibib.nih.gov/content/10-simple-rules-conformance-rubric>

**Rule 1: Define context clearly:** Develop and document the subject, purpose, and intended use(s) of the model or simulation.

**Current Conformance Level:** Comprehensive

**Model Context**: Model liver regeneration in mammals after resection.

**Primary goal of the model/tool/database:** This model was originally designed to describe the relationship between select biological signaling processes that drive liver regeneration after partial hepatectomy and the resulting mass recovery (Cook et al., 2015). In the present study, the volume recovery response of the model is compared to longitudinal liver volume recovery data measured via non-invasive ultrasound in male and female rats. Specifically, we used parameter scanning to find Metabolic Load and Cell Death Constant parameter values that best-fit data from each animal in our study in order to contextualize the observed differences in regeneration due to sex and diet.

**Key model components for additional model context:** The primary input for the model is the relative fraction of liver tissue remaining after resection, and the ordinary differential equations solve for Cell Mass and the proportion of hepatocytes in quiescent (Q_heps_), primed (P_heps_) and replicating (R_hep_) states, as well as the following molecular elements: Immediate/Early Genes, Growth Factor, Extracellular Matrix, TNF, JAK, STAT3, and SOCS3. The volume recovery curve is calculated by the equation (Q_heps_+Cell-Mass*(P_heps_+ R_heps_))+0.01. In the original publication, the model parameters were fit to liver mass recovery data in rats (Cook et al., 2015).

**Biological Domain of the Model:** Organ-scale liver regeneration in a rat model of partial hepatectomy. JAK-STAT signalling pathway, extracellular matrix. TNFa and IL-6 signalling.

**Structures of the Model**: Cells: hepatocytes. Molecules: Immediate Early Genes, Growth Factor, Extracellular Matrix, TNF, JAK, STAT3, SOCS3

**Spatial Scales Included in the Model:** All values in the model represent organ-wide abundances. Organ-scale mass/volume is relative where 1 is equal to the pre-existing liver volume (~5,000 - 10,000 mm^3^).

**Time Scales Included in the Model:** 0 to 400 hours.

**Other uses for the model (optional):** Other studies used this model to simulate virtual human patients undergoing liver resection with varying degrees of recovery (Verma et al., 2018; Verma et al., 2019).

**Rule 2: Use appropriate data:** Employ relevant and traceable information in the development or operation of a model or simulation.

**Current conformance level**: Comprehensive

| **Data for building the model** | **Published?** | **Private?** | **How is credibility checked?** | **Current Conformance Level** |
| --- | --- | --- | --- | --- |
| in vitro (primary cells cell, lines, etc.) | N/A | N/A | N/A | N/A |
| ex vivo (excised tissues) | Yes:  Tanoue, et al. 2011; Shu, et al. 2009; and Pomfret, et al. 2003 | No | the source data is confirmed to meet detailed data requirements for consistency and source description | Comprehensive |
| in vivo pre-clinical (lower-level organism or small animal) | Yes: available as supplement to the present manuscript | No | the source data is confirmed to meet detailed data requirements for consistency and source description | Comprehensive |
| in vivo pre-clinical (large animal) | N/A | N/A | N/A | N/A |
| Human subjects/clinical | Yes: Yamamoto et al., 2016 | No | the source data is confirmed to meet detailed data requirements for consistency and source description | Comprehensive |

| **Data for validating the model** | **Published?** | **Private?** | **How is credibility checked?** | **Current Conformance Level** |
| --- | --- | --- | --- | --- |
| in vitro (primary cells cell, lines, etc.) | N/A | N/A | N/A | N/A |
| ex vivo (excised tissues) | N/A | N/A | N/A | N/A |
| in vivo pre-clinical (lower-level organism or small animal) | Yes - current paper | No | the source data is confirmed to meet detailed data requirements for consistency and source description | Comprehensive |
| in vivo pre-clinical (large animal) | N/A | N/A | N/A | N/A |
| Human subjects/clinical | N/A | N/A | N/A | N/A |

**Rule 3: Evaluate within context:** Perform verification, validation, uncertainty quantification, and sensitivity analysis of the model or simulation with respect to the reality of interest and intended use(s) of the model or simulation.

**Current conformance level**: Extensive

|  | **Who Does It?** | **When does it happen?** | **How is it done?** | **Current Conformance Level** |
| --- | --- | --- | --- | --- |
| **Verification** | Developer | During development | Comparison of model output with human subjects/clinical data | Extensive - See Cook et al., 2015 and Cook et al., 2018 |
| **Validation** | Developer | During development | model was used to reproduce experimental simulations and figures | Extensive |
| **Uncertainty Quantification** | User performs uncertainty quantification | Can be performed every time the model is run for a new scenario | User discretion | Adequate |
| **Sensitivity Analysis** | Developer and User | Can be performed after every new simulation | User discretion | Extensive - Methods documented in Cook et al., 2015. |

**Rule 4: List limitations explicitly:** Provide restrictions, constraints, or qualifications for or on the use of the model or simulation for consideration by the users or customers of a model or simulation.

**Current conformance level:** Extensive

| **Disclaimer statement (explain key limitations)** | **Who needs to know about this disclaimer?** | **How is this disclaimer shared with that audience?** | **Current Conformance Level** |
| --- | --- | --- | --- |
| Limited human/rodent kinetic data for parameterization | Users | Stated explicitly in the main text | Extensive |
| Parameterization of the model is specific for human and rodent-like simulations, but model elements are identical | Users | Stated explicitly in the main text | Extensive |
| Only the JAK-STAT pathway is described explicitly by this model. All other pathway representations are simple mathematical approximations of more complex processes. | Users / Clinicians | Stated explicitly in the main text | Extensive |
| Growth factor signaling and the Immediate-Early gene expression resulting from the JAK-STAT pathway originate from a single source, and simulate hepatocyte priming in a simplified way. It has been shown that signals from many sources are able to induce hepatocellular proliferation. | Users / Clinicians | Stated explicitly in the main text | Extensive |

**Rule 5: Use version control:** Implement a system to trace the time history of modeling and simulation activities including delineation of each contributors’ efforts.

**Current Conformance Level:** Adequate

|  | **Naming Conventions?** | **Repository?** | **Code Review?** |
| --- | --- | --- | --- |
| **individual modeler** | N/A |  | Yes |
| **within the lab** | Yes | Yes | Yes |
| **collaborators** | Yes | No | Yes |

**Rule 6: Document adequately**: Maintain up-to-date informative records of all modeling and simulation activities, including simulation code, model mark-up, scope and intended use of modeling and simulation activities, as well as users’ and developers’ guides

**Current Conformance Level:** Extensive

|  | **Current Conformance Level / Target Conformance Level** |
| --- | --- |
| **Code Commented?** | Comprehensive: comments made in the model file |
| **Scope and intended use described?** | Comprehensive: described in the main text |
| **User’s Guide** | Comprehensive: described in README.txt in model repository |
| **Developer’s Guide?** | Partial: Details of development in methods of main text |

**Rule 7: Disseminate Broadly:** Share all components of modeling and simulation activities, including simulation software, models, simulation scenarios and results.

**Current Conformance Level:** Comprehensive

| **Target Audience(s):** | **“Inner Circle”** | **Scientific Community** | **Public** |
| --- | --- | --- | --- |
| **Simulations** |  |  | Description of simulations stated in the main text |
| **Models** |  |  | Model file present in supplementary material |
| **Software** |  |  | All model files and simulations run in R programming language, an open-source language, publicly and freely available |
| **Results** |  |  | Stated in main text |
| **Implication of Results** |  |  | Stated in main text |

**Rule 8: Get independent reviews:** Have the modeling and simulation activity reviewed by nonpartisan third-party users and developers.

**Current Conformance Level:** Extensive

| **Reviewer(s) name and affiliation** | **John Han (Thomas Jefferson University)** |
| --- | --- |
| When was the review performed | July 17 - 30, 2021 |
| How was the review performed and outcomes of the review? | A departmental colleague with fluency in the R programming language, not involved in the present study or liver biology research, performed the review.  Parameters were checked for consistency, simulation results were extensively evaluated |

**Rule 9: Test Competing Implementations:** Use contrasting modeling and simulation implementation strategies to check the conclusions of different strategies against each other.

**Current Conformance Level:** Adequate

|  | **Yes or No (briefly summarize)** |
| --- | --- |
| **Were competing implementations tested?** | No |
| **Did this lead to model refinement or improvement?** | The model was derived from a previously refined model that tested competing implementations (Cook et al., 2015 versus Cook et al., 2018; Verma et al., 2019). |

**Rule 10: Conform to standards:** Adopt and promote generally applicable and discipline specific operating procedures, guidelines, and regulations accepted as best practices.

**Current Conformance Level:** Extensive

|  | **Yes or No (briefly summarize)** |
| --- | --- |
| **Are there operating procedures, guidelines, or standards for this type of multiscale modeling?** | Yes, as described in the credible practice of modeling and simulation in healthcare: ten rules from a multidisciplinary perspective (Erdemir et al., 2020) |
| **How do your modeling efforts conform?** | This model is implemented in the widely used R programming language, using freely available packages. The github repository includes versions optimized for both Windows and Linux operating systems. |

**References:**

1. Erdemir, A., Mulugeta, L., Ku, J. P., Drach, A., Horner, M., Morrison, T. M., Peng, G., Vadigepalli, R., Lytton, W. W., & Myers, J. G., Jr (2020). Credible practice of modeling and simulation in healthcare: ten rules from a multidisciplinary perspective. Journal of translational medicine, 18(1), 369. https://doi.org/10.1186/s12967-020-02540-4
2. Cook, Daniel, Babatunde A. Ogunnaike, and Rajanikanth Vadigepalli. "Systems analysis of non-parenchymal cell modulation of liver repair across multiple regeneration modes." *BMC systems biology* 9, no. 1 (2015): 1-24.
3. Cook, Daniel, Sirisha Achanta, Jan B. Hoek, Babatunde A. Ogunnaike, and Rajanikanth Vadigepalli. "Cellular network modeling and single cell gene expression analysis reveals novel hepatic stellate cell phenotypes controlling liver regeneration dynamics." *BMC systems biology* 12, no. 1 (2018): 1-29.
4. Verma, Babita K., Pushpavanam Subramaniam, and Rajanikanth Vadigepalli. "Modeling the dynamics of human liver failure post liver resection." *Processes* 6, no. 8 (2018): 115.
5. Verma, Babita K., Pushpavanam Subramaniam, and Rajanikanth Vadigepalli. "Model-based virtual patient analysis of human liver regeneration predicts critical perioperative factors controlling the dynamic mode of response to resection." *BMC systems biology* 13, no. 1 (2019): 1-15
6. Tanoue S, Uto H, Kumamoto R, Arima S, Hashimoto S, Nasu Y, et al. Liver regeneration after partial hepatectomy in rat is more impaired in a steatotic liver induced by dietary fructose compared to dietary fat. Biochem Biophys Res Commun. 2011;407(1):163–8.
7. Shu R, Zhang F, Wang F, Feng D, Li X, Ren W, et al. Adiponectin deficiency impairs liver regeneration through attenuating STAT3 phosphorylation in mice. Lab Investig. 2009;89(9):1043–52.
8. Pomfret E, Pomposelli J, Gordon F, Erbay N, Price L, Lewis W, et al. Liver regeneration and surgical outcome in donors of right-lobe liver grafts. Transplantation. 2003;76(1):5–10.
9. Yamamoto, Kimiyo N., Masatsugu Ishii, Yoshihiro Inoue, Fumitoshi Hirokawa, Ben D. MacArthur, Akira Nakamura, Hiroshi Haeno, and Kazuhisa Uchiyama. "Prediction of postoperative liver regeneration from clinical information using a data-led mathematical model." Scientific reports 6, no. 1 (2016): 1-9.
