# Supplementary material for: Longitudinal ultrasound imaging and network modeling in rats reveal sex-dependent suppression of liver regeneration after resection in alcoholic liver disease
Source: Front Physiol. 2023 Mar 9;14:1102393. doi: 10.3389/fphys.2023.1102393 (PMC10033530; doi:10.3389/fphys.2023.1102393)
Supplement: Supplementary file 18 [file DataSheet1.docx]

**Supplementary File 1:** List of equations that constitute the computational model developed by Cook et al. (2015) and employed in the present study.

Model Equations:

1. $\frac{dQ}{dt}= -k_{QP} (\left[ IE \right]-\left[ {IE}_{0} \right]Q+k_{RQ}\left[ ECM \right]R+k_{req}\sigma_{req}P-k_{req}\sigma_{cd}Q$
2. $\frac{dP}{dt}= k_{QP} \left( \left[ IE \right]-\left[ {IE}_{0} \right] \right)Q-k_{PR}\left( \left[ GF \right]-\left[ {GF}_{0} \right] \right)P-k_{req}\sigma_{req}P-k_{cd}\sigma_{cd}P$
3. $\frac{dR}{dt}=k_{PR} \left( \left[ GF \right]-\left[ {GF}_{0} \right] \right)P-k_{RQ}\left[ ECM \right]R+k_{prol}R-k_{cd}\sigma_{cd}R$
4. $\frac{d[IL6]}{dt}=k_{IL6}\frac{M}{N+ ɛ}-\frac{V_{JAK}\left[ IL6 \right]}{\left[ IL6 \right]+K_{M}^{JAK}}-\kappa_{IL6}\left[ IL6 \right]+k_{1}$
5. $\frac{d[JAK]}{dt}=\frac{V_{JAK}\left[ IL6 \right]}{\left[ IL6 \right]+K_{M}^{JAK}}-\kappa_{JAK}\left[ JAK \right]+k_{2}$
6. $\frac{d[STAT3]}{dt}=\frac{V_{ST3}\left[ JAK \right]{[proSTAT3]}^{2}}{\left[ proSTAT3 \right]^{2}+K_{M}^{ST3}(1+\frac{\left[ SOCS3 \right]}{K_{I}^{SOCS3}})}-\frac{V_{IE}\left[ STAT3 \right]}{\left[ STAT3 \right]+K_{M}^{IE}}{-\frac{V_{SOCS3}\left[ STAT3 \right]}{\left[ STAT3 \right]+K_{M}^{SOCS3}}-\kappa}_{ST3}\left[ STAT3 \right]+k_{3}$
7. $\frac{d[SOCS3]}{dt}={\frac{V_{SOCS3}\left[ STAT3 \right]}{\left[ STAT3 \right]+K_{M}^{SOCS3}}-\kappa}_{SOCS3}\left[ SOCS3 \right]+k_{4}$
8. $\frac{d[IE]}{dt}=\frac{V_{IE}\left[ STAT3 \right]}{\left[ STAT3 \right]+K_{M}^{IE}}{-\kappa}_{IE}\left[ IE \right]+k_{5}$
9. $\frac{d[GF]}{dt}=k_{GF}\frac{M}{N+ ɛ}{-k_{up}[GF][ECM]-\kappa}_{GF}\left[ GF \right]+k_{7}$
10. $\frac{d[ECM]}{dt}={-k}_{deg}\left[ IL6 \right][ECM]{-\kappa}_{ECM}\left[ ECM \right]+k_{6}$
11. $\frac{dG}{dt}=k_{G}\left( \frac{M}{N+ ɛ} \right)-{-k}_{G}M$

Where:

1. $ɛ=0.01$
2. $\sigma_{cd}= 0.5(1+\tanh(\frac{\theta_{cd}-(N+ɛ)/M}{\beta_{cd}})$
3. $\sigma_{req}= 0.5(1+\tanh(\frac{\theta_{req}-[GF]}{\beta_{req}})$
4. $N=Q+G(P+R)$
5. $k_{1}=\frac{V_{JAK}}{1+K_{M}^{JAK}}-k_{IL6}\frac{M}{N_{ss}+ ɛ}{+\kappa}_{IL6}$
6. $k_{2}=\kappa_{JAK}-\frac{V_{JAK}}{1+K_{M}^{JAK}}$
7. $k_{3}=\frac{V_{ST3}{[proSTAT3]}^{2}}{\left[ proSTAT3 \right]^{2}+K_{M}^{ST3}(1+\frac{1}{K_{I}^{SOCS3}})}+ \frac{V_{IE}}{1+K_{M}^{IE}}+\frac{V_{SOCS3}}{1+K_{M}^{SOCS3}}{+\kappa}_{ST3}$
8. $k_{4}=\frac{V_{SOCS3}}{1+K_{M}^{SOCS3}}{+\kappa}_{SOCS3}$
9. $k_{5}=\frac{V_{IE}}{1+K_{M}^{IE}}{+\kappa}_{IE}$
10. $k_{6}=k_{deg}{+\kappa}_{ECM}$
11. $k_{7}=-k_{GF}\frac{M}{N_{ss}+ ɛ}{+k_{up}+\kappa}_{GF}$
12. $N_{SS}=0.99$
13. $Q_{0}=remnant liver fraction; P_{0}=0; R_{0}=0;G_{0}=1; \left[ {IL6}_{0} \right]=1; \left[ {JAK}_{0} \right]=1; \left[ {STAT3}_{0} \right]=1; \left[ {SOCS3}_{0} \right]=1; \left[ {IE}_{0} \right]=1; \left[ {GF}_{0} \right]=1; \left[ {ECM}_{0} \right]= 1; N_{0}=Q_{0}+G_{0}\left( P_{0}+R_{0} \right)= Q_{0}$

**References:**

Cook, Daniel, Babatunde A. Ogunnaike, and Rajanikanth Vadigepalli. "Systems analysis of non-parenchymal cell modulation of liver repair across multiple regeneration modes." *BMC systems biology* 9, no. 1 (2015): 1-24.
